# Adolescent mental health and social inequality in the aftermath of COVID-19 in Bogotá, Colombia: a qualitative study using a critical ecological model

**DOI:** 10.1186/s12889-026-26293-9

**Published:** 2026-01-21

**Authors:** Johanna Carolina Sánchez-Castro, Nelly Esther Caliz Romero, Laura Pilz González, Christiane Stock, Katherina Heinrichs

**Affiliations:** 1 Institute of Health and Nursing Science, Charité – Universitätsmedizin Berlin, corporate member of Freie Universität Berlin and Humboldt-Universität zu Berlin, Augustenburger Platz 1, Berlin, 13353 Germany; 2 Facultad de Enfermería, Universidad Nacional de Colombia, Carrera 45 N° 26-85 Edificio 228, Bogotá, 111321 Colombia

**Keywords:** Adolescents, Mental health, Social inequality, Family, Peer relationships, Urban contexts, COVID-19 pandemic, Gender, Ecological model.

## Abstract

**Background:**

Adolescence is a stage of life characterised by significant biological, social, and emotional changes, making it a particularly challenging period for mental health. Young people face these challenges within environments shaped by social, cultural, and economic dynamics, often rooted in structural inequality. In urban contexts such as the megacity of Bogotá, many adolescents live in conditions of social inequality. The COVID-19 pandemic intensified pre-existing issues and introduced new challenges to adolescent mental health. This study explores the mental health of adolescents living in an urban setting characterised by social inequality, including both the benefits and challenges associated with the COVID-19 pandemic.

**Methods:**

Using a qualitative approach, direct observations were conducted in public schools and socially vulnerable neighbourhoods in Bogotá. Complementary problem-centred interviews were carried out with adolescents and school counsellors at three public schools in these areas. The interviews focused on topics related to social inequality, mental health, and key experiences during the COVID-19 pandemic. A reflexive thematic analysis was conducted. The research adhered to national and international ethical guidelines.

**Results:**

A total of 95 h of field observation were conducted, alongside 42 interviews with adolescents aged 12–18 (57% female) and six school counsellors, most of whom were female psychologists. The findings are presented through the the Critical Ecological Model of Adolescents’ Mental Health, which illustrates adolescents’ mental health and its changes concerning the pandemic across four levels: (a) personal: self-care, life plans, emotional distress, and post-pandemic coping; (b) interpersonal: family and peer relationships; (c) community: support networks, disadvantage, and violence; and (d) organisational/media: school-based support and mental health promotion and care.

**Conclusions:**

In urban settings marked by social inequality, supportive environments within families, schools, communities, and institutions are essential. Engaging with adolescents’ perspectives highlights both their needs and their capacities to navigate challenges and support mental health across different contexts. Structural strategies are required to confront social inequalities and promote both individual and collective actions to support adolescent mental health, particularly in post-pandemic contexts.

**Supplementary Information:**

The online version contains supplementary material available at 10.1186/s12889-026-26293-9.

## Introduction

Adolescence is widely recognised as a crucial stage of human development, marked by profound biological, physical, cognitive, emotional, and social changes, the effects of which often extend into adulthood [1]. This period encompasses a range of developmental tasks, including the formation of personal identity, the acquisition of emotional regulation skills, and the establishment of social relationships beyond the family unit [2, 3]. Adolescents navigate complex social expectations while exploring autonomy, negotiating peer relationships, and asserting independence [4, 5]. They engage in identity experimentation, develop future aspirations, and cultivate a sense of belonging within wider social networks [6]. During this time, experiences are deeply shaped by family dynamics, peer interactions, educational settings, and broader socio-cultural contexts [3, 6]. How adolescents are able to navigate these challenges under conditions that are unequally distributed may contribute to the development of resilience, self-efficacy, and emotional competence. Conversely, sustained exposure to conflict, neglect, or structurally produced social disadvantage can constrain these processes and increase vulnerability to mental health problems. Importantly, these processes should not be understood as individual achievements but as relational and context-dependent outcomes shaped by structural inequalities [3, 6].

Mental health during adolescence is a core component of overall well-being and essential for healthy development and social integration [6]. It encompasses the capacity to manage daily challenges, sustain positive relationships, and realise personal potential [7]. Emotional development involves recognising and regulating one’s feelings, cultivating empathy, and responding to others’ emotional needs, while social development includes expanding peer networks, exploring roles and identity, and negotiating belonging within communities [6, 8, 9]. The interaction between individual capacities and environmental influences, including family, school, community, and broader social structures, shapes opportunities for growth and resilience [3, 6]. Supportive, safe, and nurturing environments enable adolescents to strengthen coping strategies, develop self-confidence, and maintain psychosocial balance [10–12]. In contrast, exposure to social inequity, family conflict, or unsafe contexts can compromise mental health, underlining the importance of structural and relational factors in promoting well-being and preventing maladaptive outcomes during this formative stage [13]. Structural social characteristics, such as power relations, the production and accumulation of wealth, and social relations shaped by intersecting sociocultural identities, including ethnicity/race, gender, and class, influence mental health [14–16].

Social inequality is a persistent and pervasive feature in several countries in Latin America, including Colombia [17]. In urban contexts like Bogotá, young people’s everyday lives are marked by structural inequalities that manifest in poverty, precarious housing, overcrowding, and limited access to quality education [18, 19]. Unequal opportunities for recreation, cultural engagement, and safe public spaces further restrict their possibilities for healthy development [18, 19]. These conditions not only generate environments of stress, family and community conflict, insecurity, and exposure to violence, but also reinforce cycles of disadvantage that hinder adolescents’ well-being and quality of life [16, 20]. These influences can be conceptualised through the Mental Health and Well-being Ecological Model, which highlights the intersecting and cumulative relationships across different social contexts and how they collectively shape adolescent mental health [21]. The levels stretch from the individual level up to the societal level which also influences adolescents’ mental health, especially in the time of crises [19].

Within this already unequal social landscape, young people were faced with an unprecedented global event: the COVID-19 pandemic and the public health measures implemented to contain its spread [2]. Among these, mandatory lockdowns and the sudden shift to remote learning had the most direct impact on adolescents’ daily lives. Between 2020 and 2021, many spent between 12 and 18 months confined to their homes, with limited opportunities for in-person social interaction [22, 23]. This prolonged period of confinement increased the amount of time spent with family members while reducing access to peers and external support networks [24]. These dramatic changes significantly affected adolescents’ mental health, intensifying existing challenges and generating new stressors within both the home and the broader social environment [25]. Prolonged lockdowns did not simply produce temporary disruptions but exacerbated long-standing processes of social and economic precarisation that function as direct structural determinants of adolescent mental health, particularly in highly unequal urban settings [26, 27].

Despite the growing recognition of adolescence as a critical stage for mental health, there remains a limited understanding of how structural inequalities in urban settings shape young people’s mental health, particularly in contexts marked by social inequality and the disruptions brought about by the COVID-19 pandemic. In this study, social inequality is not conceptualised as a contextual backdrop, but as an active force shaping adolescents’ living conditions, subjectivities, relationships, and possibilities for action, with enduring consequences for their mental health trajectories.

Although post-pandemic research on adolescent mental health has expanded, the existing literature in Latin America has largely prioritised quantitative designs and short-term outcome measures, often focusing on individual risk factors or diagnostic categories. Consequently, in-depth qualitative studies remain scarce. Such studies could focus on adolescents’ own perspectives and examine how young people from low-income urban contexts experience, interpret, and navigate structurally produced inequalities in their everyday lives. In Bogotá in particular, situated qualitative evidence foregrounding adolescents’ voices remains limited.

By drawing on Latin American Social Medicine and the Social Determination of Health framework, this study addresses this gap. We offer a contextually grounded analysis of adolescent mental health that speaks not only to the Colombian case but also to broader regional dynamics of inequality, urban precarisation, and institutional fragility. In doing so, we seek to contribute not only to academic knowledge but also to more just and socially grounded approaches to promoting adolescent mental health. Accordingly, this study aims to explore the mental health experiences of adolescents living in an urban setting characterised by social inequality, examining both the challenges and perceived benefits associated with the COVID-19 lockdown.

## Methods

### Study design and setting

This study adopts a qualitative constructivist approach, understanding adolescent mental health as both a social and personal construction shaped by their familial, community, and structural contexts [28]. In line with a Big Q qualitative orientation, the research used reflexive thematic analysis to explore the meanings that adolescents themselves attribute to their emotions [29]. The study was conducted in Bogotá, the capital city of Colombia, which exhibits a complex interplay of social, economic, and cultural dynamics, making it a suitable setting for research in urban contexts [18]. Three public secondary schools agreed to participate, all located in the districts of Bosa and Tunjuelito. These areas are characterised by low Basic Quality of Life Index scores and poverty rates that exceed the city average of 4.3% (Bosa: 11.4% and Tunjuelito: 9.6%) [18, 30, 31].

The fieldwork was conducted by JCSC between October 2022 and June 2023, using two complementary methods: direct observation and problem-centred interviews. In both cases, structured guides were developed specifically for this study to facilitate systematic data collection (see Additional files 1, 2 and 3). JCSC drafted the initial versions of these tools, which were then discussed and refined with each member of the research team (NECR, LPG, KH, and CS). This study received ethical approval from Charité – Universitätsmedizin Berlin (application number EA2/056/22, date of approval 09.05.2022) and the Secretaría de Salud Distrital - Bogotá (project code SDSCTI20220011, date of approval 19.10.2022). All procedures were performed in accordance with the ethical standards of the institutional and local research committees and with the principles of the Declaration of Helsinki. As a guide for reporting the results of this study, we used the Standards for Reporting Qualitative Research (SRQR) [32].

### Participants and recruitment

Interviewees were recruited using school-based strategies. Adolescents were invited to take part through the distribution of leaflets within schools, which included an overview of the study. Those who expressed interest received an informed consent form, which required both their own signature and that of a parent or legal guardian, in line with Colombian regulations and the requirements of the ethics committees. Written informed consent was always obtained prior to conducting the interviews. School counsellors were contacted directly in their workplaces, received detailed information about the project, and were asked if they wished to participate; those who consented also signed an informed consent form. Participant recruitment followed a purposive sampling strategy, complemented by elements of snowball sampling [33]. Eligible participants included adolescents aged between 12 and 18 years, regardless of gender, enrolled in the three participating public schools in Bogotá, as well as counsellors of any age or gender working in the same institutions and engaged in adolescent (mental) health promotion. There were no exclusion criteria. The sample size was determined based on the concept of information power, which suggests that the greater the information power held by the sample, the fewer participants are required [34].

### Direct observation

A non-participant direct observation technique was employed, avoiding intentional influence on adolescents’ behaviour, to closely observe and listen to everyday situations potentially related to the research question [35, 36]. During the observations, JCSC walked through school corridors and courtyards or sat among adolescents during classes, recess, and meetings with coordinators, teachers, and/or parents. At times, she engaged in casual conversations with those around her. Although participants were not explicitly informed that they were being observed, when asked, JCSC clearly and briefly explained her role as an observer.

To gain a deeper understanding of the adolescents’ broader living context, JCSC also conducted observations in the neighbourhoods mentioned during the interviews, generally located within the same or adjacent districts to the schools. For safety reasons, these observations were carried out from a car or motorcycle, always accompanied, and involved driving through streets or around local parks. Field observations were consistently recorded in a field notebook, capturing not only descriptions of interactions and dialogues relevant to the research focus but also the researcher’s impressions, initial interpretative insights, and theoretical reflections.

### Interviews

Problem-centred interviews were conducted based on a semi-structured interview guide, in order to obtain detailed and in-depth information about the experiences, thoughts, and emotions of the participants [37–39]. Prior to beginning each interview, a brief standardised questionnaire was verbally administered to collect participants’ demographic details, with responses manually recorded by the interviewer. For adolescent participants, the data gathered included age, gender, place of origin, household composition, primary caregiver, parental educational attainment, housing conditions, and access to digital resources such as home internet connectivity. For counsellors, information regarding gender and professional background was documented. These data were collected to provide a basic demographic profile of participants and contextualise their narratives.

To create a relaxed atmosphere, adolescents were initially invited to describe a typical day in their lives. The subsequent interview focused on several key topics, each introduced by a core question and complemented with optional prompts: (a) understanding of social inequality; (b) emotional reactions to inequality; (c) awareness of public health measures related to COVID-19; (d) perceived influence of these measures and inequality on adolescents’ lives; (e) general views on mental health; and (f) perceived changes in adolescent mental health concerning social inequality and pandemic restrictions. Interviews with school counsellors began with a broad inquiry about their job and daily activities with adolescents, after which they were asked to consider the same thematic areas, drawing on their observations and experience working with adolescents. At the conclusion of each interview, all participants were invited to contribute any additional insights or reflections they felt were relevant. All interview questions were open-ended, encouraging participants to speak freely and reflectively [40].

All interviews were conducted in Spanish, the primary language of both participants and the interviewer, and took place in person in quiet locations provided by the school, including unused classrooms, meeting areas, or counselling offices. These settings were selected to promote a secure, comfortable, and confidential environment, following ethical research standards. A single audio recording device, incapable of connecting to the internet, was used consistently for all interviews.

### Data analysis

A reflexive thematic analysis was conducted at a latent level, aiming to move beyond the surface meaning of participants’ accounts to construct patterns of meaning that reflect underlying assumptions, social mechanisms, and contextual influences shaping adolescents’ mental health [29, 41, 42]. During the initial phases of analysis, an inductive orientation guided the coding process, in which codes were actively constructed from the data without applying predefined theoretical frameworks [29, 41, 42]. In the later stages, a deductive orientation informed the interpretation and organisation of themes. That is, while the themes were generated inductively in relation to participants’ narratives, theoretical concepts were subsequently used to interrogate the relationships between them and to develop a coherent analytical narrative [29, 41, 42]. This hybrid approach facilitated a grounded yet theoretically informed understanding of adolescents’ experiences of mental health.

The analytical process followed six interconnected phases [29, 41, 42]. Initially, all field notes were systematically compiled and arranged in chronological order. Each interview was assigned a unique identifier by JCSC (e.g., A + number for adolescents and C + number for counsellors). Transcriptions were ‘transcribed verbatim and carefully verified against the audio recordings to correct inaccuracies and remove any potentially identifying information. All quotes used to illustrate the results are also presented in the original language (Spanish) in the Additional file 4. For the main text, quotes were initially translated using ChatGPT (OpenAI, 2025) and subsequently revised and refined by a bilingual (LPG) researcher (native Spanish speaker, fluent in English) to ensure semantic accuracy, contextual fidelity, and preservation of participants’ intended meanings. Final versions of all translated excerpts were reviewed, validated, and approved exclusively by the research team, who retain full responsibility for the accuracy and integrity of the translated material.

In the second phase, JCSC engaged deeply with the dataset, generating analytically rich codes (concise, interpretative labels that captured features of the data relevant to the research question). A subset of the dataset (approximately a quarter) was independently coded by JCSC and NECR. The research team pragmatically determined this proportion to stimulate dialogue, facilitate critical discussion of interpretations and enhance reflexivity throughout the analytic process. This subset included interviews and field notes identified as particularly salient at the time. This collaborative exercise supported the development of a provisional coding framework, which remained open to iterative revision [29].

In the third phase, JCSC applied and further refined this coding framework across the entire dataset. The process remained interpretative and flexible, allowing for the construction of new codes and the reorganisation of existing ones in response to analytical insights [29].

In the fourth phase, codes were actively clustered to construct candidate themes (conceptual groupings that captured patterned meaning across the dataset in relation to the research focus). This was an iterative and interpretative process involving continual engagement with codes to ensure internal coherence and clear boundaries between developing themes [29].

The fifth phase entailed a comprehensive re-examination of the dataset to evaluate the thematic structure’s analytical robustness. This interpretative work was informed by the Mental Health and Well-being Ecological Model, which visually illustrates how structural, collective, and individual processes interact to shape mental health outcomes [21]. Visual mapping techniques were employed to explore thematic interrelations and support conceptual clarity [29].

In the sixth and final phase, each theme and subtheme was carefully reviewed, revised, and consolidated to ensure conceptual clarity, coherence with the dataset, and alignment with the research questions. Names were carefully developed to reflect their conceptual scope and analytical intent. JCSC held regular meetings with other members of the research team (NECR, LPG, KH, and CS), which supported analytical rigour and reflexivity. These collaborative exchanges helped review and sharpen the limits, associations, and labels of the thematic structure [29]. Finally, JCSC crafted the analytic narrative, integrating illustrative data extracts to bring conceptual patterns to life. This narrative was reviewed and refined in consultation with the wider team. The thematic analysis was supported using the software MAXQDA 24.0.0 (VERBI Software, Berlin, Germany).

### Reflexivity

JCSC has long-standing familiarity with the research setting, having grown up in the area where the fieldwork took place (Bosa). Although her schooling experience differed from that of local public schools, her close personal connections with these institutions provided valuable contextual insight into the community dynamics studied. This background allowed JCSC to recognise, through lived experience and close relational ties, many of the structural challenges addressed in the study. JCSC trained as a nurse at a public university in Bogotá, which further grounded her understanding of the health needs and social inequalities faced by vulnerable populations. Conducting the fieldwork as part of her PhD research offered an opportunity to revisit these familiar communities from a dual position, as both insider and outsider. This perspective enabled her to approach the participants’ narratives with a mix of empathy, cultural understanding, and analytical distance, enriching both the depth of data collection and its interpretation.

The wider research team contributed disciplinary and experiential diversity to the study, bringing perspectives from nursing, psychology, as well as public and global health. Collectively, they had extensive experience working on adolescent and youth mental health in various contexts. At the time of the study, LPG was a doctoral candidate, KH a postdoctoral researcher, NECR an associate professor, and CS a full professor. Their complementary backgrounds supported critical reflection throughout the research process and strengthened the reflexivity of the study.

## Results

The fieldwork began with 95 h of direct observation across 23 sessions, carried out in the three participating schools and in the neighbourhoods where the adolescent participants lived, as identified during the interviews. This was followed by 48 semi-structured interviews. Characteristics of the sample are presented in Table 1. A total of 42 participants were adolescents aged 12 to 18, the majority being female (57%), heterosexual (79%), and born in Colombia (93%). Most lived in single-parent households, primarily under the mother’s care, and their caregivers typically had completed secondary education. The majority resided in rented apartments with internet access. In addition, six school counsellors were interviewed; these were primarily female psychologists affiliated with the schools. Observations and interviews were conducted on weekdays between October 2022 and June 2023, excluding the school holiday period at the end of December and the beginning of January.

**Table 1:** Participants’ demographics (*n* = 48)

Details		Number
*Adolescents* (*n* = 42)	*Age in years*	
	12	5
	13	7
	14	7
	15	7
	16	10
	17	3
	18	3
	*Gender*	
	Female	24
	Male	18
	Type of family*	
	Single parent	13
	Extensive	10
	Nuclear	10
	Composite	9
	*Main caregiver*	
	Mother	25
	Mother and father	11
	Father	5
	Grandmother and father	1
	*Highest schooling of primary caregivers*	
	5 grade	3
	7 grade	1
	8 grade	2
	9 grade	8
	High school	18
	Technician training	7
	Bachelor	3
*Counsellors* (*n* = 6)	*Gender*	
	Female	5
	Male	1
	*Profession*	
	Psychologist	5
	Special educator	1

*Single parent: one caregiver; Extended: includes members of different generations (e.g., grandparents, aunts/uncles); Nuclear: two parents and their children; Composite: includes multiple nuclear units or stepfamilies (e.g., mother, stepfather, and children)

**Fig. 1:**
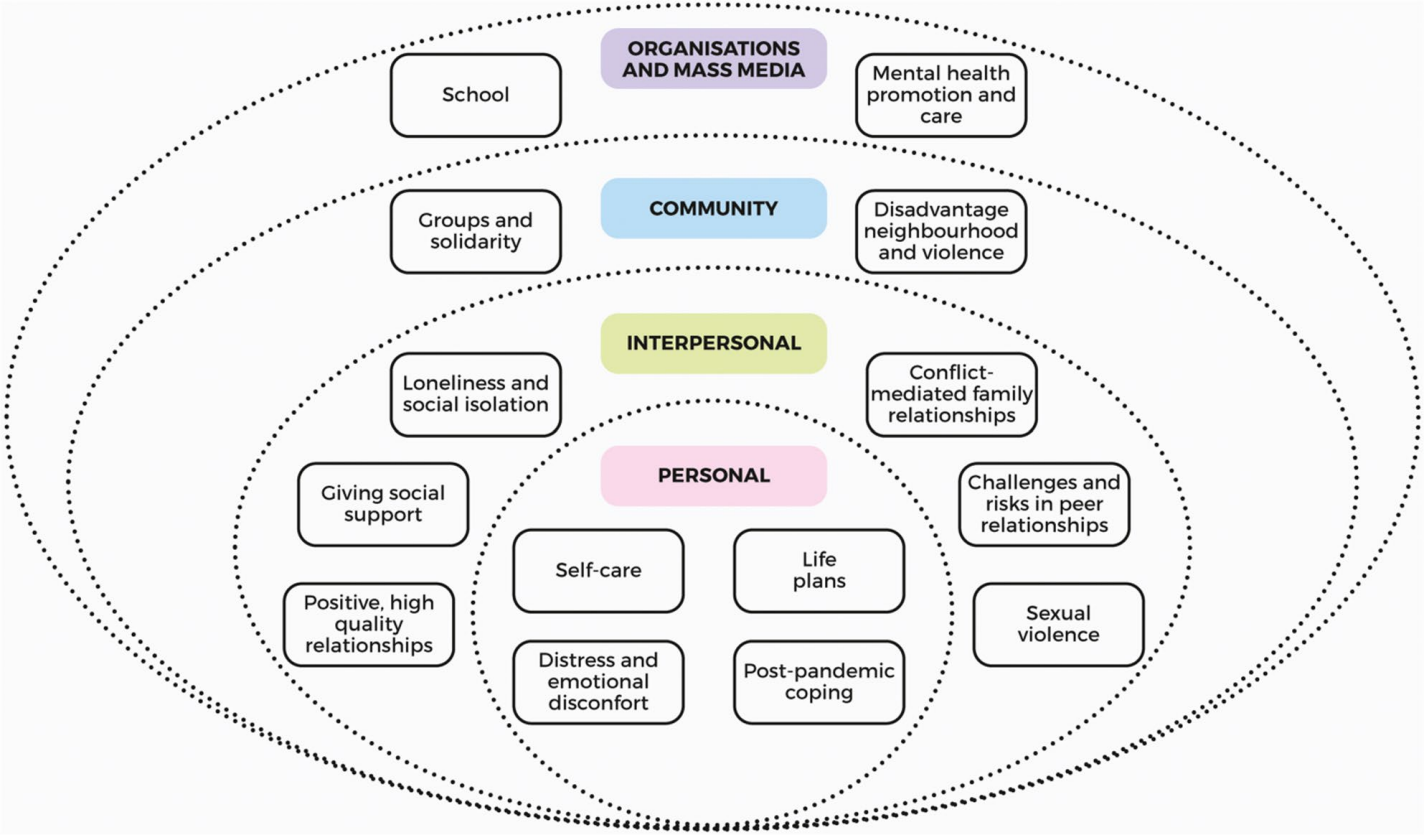
Critical Ecological Model of Adolescents’ Mental Health based on Mental Health and Well-being Ecological Model [21] (designed on miro.com)

Results from the reflexive thematic analysis led to the design of the the Critical Ecological Model of Adolescents’ Mental Health, which organises adolescents’ mental health experiences into four interconnected levels: (a) personal, (b) interpersonal, (c) community, and (d) organisations and mass media. This model was specifically developed for this study as an adaptation of the previously established Mental Health and Well-being Ecological Model [21]. Subthemes were further elaborated within each level to provide a more detailed account of adolescents’ experiences (see Fig. 1).

### Personal level

We begin by navigating the personal level of adolescent mental health, focusing on individual characteristics such as identity, self-awareness, emotional regulation, and behavioural responses. Within this level, we explore how adolescents engage in self-care, formulate life plans, deal with emotional distress, and adapt in the aftermath of the COVID-19 lockdown, processes that are deeply influenced by their evolving sense of self and autonomy.

#### Self-care

Adolescents described personal growth through improved emotional awareness and regulation, which helped them lead calmer lives with fewer or less intense episodes of stress and loss of control. They valued focusing on positive aspects of life to foster encouragement and enjoyment in daily interactions, e.g. through spirituality as a source of tranquillity. Many sought to strengthen self-perception by improving self-concept and developing a body image with which they felt comfortable. Several took pride in resisting drug use despite availability and appreciated spaces that allowed free self-expression, moments of solitude for reflection, and distance from family conflict or academic pressure.

*A37 (17 years old*,* female): Alone*,* alone*,* umm*,* well*,* it’s not that I have a big ego*,* but I also used to say to myself*,* but I’m not ugly*,* I mean*,* I don’t know*,* so what if that girl has a better body or because she’s chubby or more whatever*,* you know? Umm*,* yeah*,* like*,* I started to recover that confidence on my own. So I would ask my mom*,* “Oh*,* buy me some makeup*,* then*,* ah*,* buy me this*,*” and I started dressing nicely*,* feeling pretty*,* you know? Especially my face*,* because like*,* my body isn’t something I love*,* but it doesn’t bother me that much.*

The capacity to express emotions in safe, respectful spaces was identified as key for emotional management. While peer relationships were valued, support and guidance from primary caregivers and significant adults were seen as essential. Engagement in various activities supported emotional regulation, including sports, dance, music, painting, reading, writing, and social media use. Playing video games, spending time with pets, and relaxing, whether resting, watching television, or breaking routine with a walk or shopping trip, were also described as meaningful ways to manage emotions and leisure time.

*A11 (15 years old*,* male): For example*,* with all the problems I had last year*,* my safe space was volleyball*,* playing*,* you know what I mean? That’s what gave me peace*,* it was the only thing that really helped me with my problems.*

Both adolescents and school counsellors reflected on how the COVID-19 lockdown provided an opportunity for self-exploration and increased emotional awareness among young people. Adolescents used the additional time to strengthen themselves, clarify aspirations, and reflect on desired relationships with family and friends. Many reported discovering previously unrecognised values, abilities, and strengths, leading to improved self-perception and self-esteem. Several adolescents developed new interests during lockdown, including reading, painting, writing, playing instruments, crafting, cooking, or learning languages, activities that became meaningful alternatives during this period. Others identified strategies to break the monotony, such as watching films or series with their family, playing board or video games, or visiting relatives in other cities thanks to remote learning and work. Improvements in personal habits were also noted, including physical self-care (e.g. hair and skin care) and engaging in regular physical activity.

*A14 (15 years old*,* male): Like*,* rest*,* mental rest […] When I was at school*,* I barely had time for myself*,* to relax. However*,* this pandemic*,* even though the amount of rest time was exaggerated*,* also helped me a little to reflect on my behaviour*,* on who I was*,* and how I also wanted to give myself time for myself*,* which I didn’t do before.*

#### Life plans

Achieving educational milestones, such as completing secondary school, entering university, and becoming professionals, was commonly seen as a key strategy by adolescents for improving their living conditions. These aspirations often reflected both a desire to surpass their parents’ typically limited educational and financial backgrounds and to fulfil familial expectations. Personal experiences of economic hardship frequently shaped adolescents’ life projects, motivating them to imagine a future in which such difficulties were absent. For some, these aspirations were rooted in long-term goals requiring commitment, perseverance, and dedication; for others, they manifested in more immediate desires, such as dreams of winning the lottery. In certain cases, the urgent need to generate income led adolescents to deprioritise education in favour of informal or low-skilled work, military service, monetised content creation on social media, or seeking financially stable partners, sometimes significantly older. For another group, migration to other countries was perceived as the most viable route to financial security, offering improved employment prospects, better access to higher education, and opportunities for cultural exploration. Having a family was not a primary goal for most participants.

*A22 (13 years old*,* female): To be independent*,* not depend on anyone*,* be a source of pride for my parents. And in the future*,* to be able to be someone in life*,* you know? Like*,* for example*,* when I grow up*,* to be able to give to my parents and thank them and make them feel good*,* and for my part*,* to be on my own*,* to be able to do the things I like and all that*,* and to study what I like.*

#### Distress and emotional discomfort

Several adolescents described experiencing persistent low mood, including sadness, hopelessness, uncontrollable crying, and irritability. These emotional difficulties often arose in response to family conflicts, bereavement, breakups, or relocations. During the COVID-19 lockdown, these symptoms were exacerbated by prolonged monotony, lack of privacy, as well as pandemic-related anxieties, including fear of illness or death of loved ones and panic buying. For some, emotional dysregulation escalated into psychosomatic responses such as fainting, vomiting, abdominal pain, and severe headaches, occasionally requiring hospitalisation.

*A34 (16 years old*,* male): And well*,* let’s say I wouldn’t if I could tell you whether I’m truly happy*,* because let’ say sometimes I am*,* I won’t lie*,* I am happy sometimes when we’re joking around or laughing at some memes or silly things we see. But happy when I’m with someone or something*,* no*,* personally I wouldn’t categorise myself as someone who’s happy.*

Participants frequently reported a decline in motivation and energy, often describing their daily routines as dull and repetitive. During the persisting lockdown, initial engagement with leisure activities faded, replaced by boredom and detachment. This disengagement extended to schoolwork and self-care, leading some adolescents to neglect hygiene, decline meaningful conversations with family and peers, or lose interest in previously valued goals such as academic achievement or personal development. Feelings of emptiness and despair were common. Some adolescents reported a diminished interest in tackling problems such as financial, academic, or personal difficulties, viewing them as irrelevant.

*A9 (17 years old*,* female): I think at that time I reached a certain point of depression*,* because it really got to me*,* being locked up for so long*,* so much time*,* because those four walls*,* of course*,* drove me crazy and I was feeling bad every day… it even got to a point where for a week I didn’t get out of bed*,* I was really not well*,* I hardly ate until… I said to myself*,* I can’t let this defeat me because I have to*,* well*,* move up to tenth grade to be able to go on to eleventh.*

Experiences of diminished self-worth were often linked to external evaluations, with comments on adolescents’ roles as children, students, partners, or friends, contributing to reduced confidence. Some participants internalised these judgements, referring to themselves with terms such as “lazy” or “bad people”. Social media also influenced self-image through comparisons with others and the positive reinforcement gained from “likes” or comments on posts. Changes in eating habits during the lockdown affected body image, prompting some to avoid social interaction or conceal perceived flaws using masks.

*A42 (18 years old*,* female): You see*,* I haven’t really figured out what makes me happy. What I do know is that nothing truly makes me happy. Like*,* whatever I do*,* I don’t feel satisfied with myself […] I feel satisfied when I take a photo and it turns out nice and other like it on social media […] but I’ve come to realise that’ really bad*,* you know*,* doing things for other people instead of thinking about myself*,* so no*,* so far I haven’t really figured out something that genuinely makes me happy.*

Catastrophic thoughts and pessimistic outlooks on the future were described by participants, often linked to the sudden lifestyle disruptions and uncertainty triggered by the pandemic. Bereavement during this period was especially traumatic, given the suddenness of the loss, the absence of farewell rituals, and the significant emotional and economic burden placed on families.

*A4 (female*,* 15 years old): I feel like everything is harder now [after the pandemic]*,* uglier*,* like this is reality. Before*,* it was like “Oh*,* this is so nice*,*” kind of like a little fairytale*,* so to speak. Now it’s all just reality*,* like “Oh*,* life isn’t like that*,*” so why would I get my hopes up for something that’s not going to happen? […] the pandemic made us realise that very serious things were happening in the world.*

Self-injurious behaviours without suicidal intent emerged as a concern in the interviews and observations. Adolescents presented various forms of self-harm, such as cutting the skin (sometimes scars were visible), biting their lips until they bled, scratching excessively, using hygiene tools to inflict pain, or banging their heads against walls, as strategies to relieve intense emotional discomfort. These acts were often linked to difficulties in managing interpersonal tensions, frustration, or personal difficulties.

*A42 (18 years old*,* female): I hurt myself […] I was in the bathroom having a shower and using those rough scrubbing pads*,* I’d grab my legs and scrub them really hard*,* and at that moment*,* I felt good. But now*,* thinking back*,* I know that wasn’t okay at all. Because of that*,* I couldn’t sleep*,* I just didn’t sleep. I also started biting my lip*,* and I had this sore on my lip that bled*,* so I had to take antibiotics.*

Additionally, adolescents’ accounts reveal instances of suicidal ideation, such as fantasising about death or considering where to obtain medication to end their lives through an overdose. Suicide threats were also reported, including telling a parent or partner about intentions to die. Some described suicidal gestures, such as ingesting non-lethal medication or attempting to hang themselves with materials incapable of causing real harm, while others recounted actual suicide attempts, including jumping from a second floor or attempted hanging. Most adolescents stated that they faced these experiences alone, and for many, this was the first time they had disclosed these thoughts or behaviours due to fear of being judged. Counsellors confirmed that such behaviours were commonly reported in their daily work with students.

*A4 (female*,* 15 years old): There was a moment when I actually thought about ending my life because I said*,* I don’t want to exist anymore*,* if I’m not good for my mum or my siblings*,* then I don’t want to exist. But then she [my girlfriend] started telling me that I couldn’t leave her alone and that it couldn’t happen because it [being lesbian] was something that*,* eventually*,* would become normal for my mum and things like that… and I thought*,* well*,* I have to wait.*

During the lockdown, stressors such as family conflicts, academic frustrations, loneliness, and lack of motivation intensified, leading to the onset of self-harming and suicidal behaviours previously absent. One adolescent reported cutting her arm, and two others described suicidal gestures during confinement.

*A7 (15 years old*,* female): Yes*,* that was also during the pandemic because*,* first*,* there was the pressure from school*,* they gave us too much homework […] my parents always arguing*,* sometimes they fought really badly and said horrible things to each other […] I did think about it once*,* at that time when everything was so awful*,* and I said to myself*,* I know how to do it*,* I’ll grab a knife from the kitchen […] I was actually planning it*,* thinking I could do this*,* until I realised*,* what am I doing? What am I thinking? Am I really thinking about dying? Once I did actually pick up a knife*,* and obviously I didn’t go through with it*,* but I did end up leaving a mark…*.

#### Post-pandemic coping

Upon returning to school after 18 to 24 months of confinement and virtual classes, adolescents reported various reactions. Some adolescents returned to school with a focus on reconnecting with old friends and sharing new experiences, or on building new friendships after losing contact with previous peers during the lockdown period. Others returned with the goal of improving academic performance, and sustaining habits built during the pandemic. They noted that rapid changes taught them to be more flexible and to approach challenges calmly. They expressed greater appreciation for family, friends, and the ability to go out safely, feeling more aware of the value of these previously overlooked aspects. They also identified pandemic-related benefits, such as improved hygiene, use of technology, and seeing the pandemic as a challenge they had overcome.

*A17 (12 years old*,* female): So*,* I started to think that I should value time better. Like*,* I’ve really begun to appreciate more the time I spend with someone I like*,* someone I get on well with. My perspective on life has really become clearer*,* it’s about finding a purpose and at least fulfilling one dream.*

For others, the return was unsettling. They noticed physical changes in peers, leading to body image dissatisfaction, and felt overwhelmed by the need to socialise after forgetting how to do so during confinement. Social anxiety hindered their participation in activities and forming new connections. Re-adapting to early mornings, long days, and heavy workloads was stressful after pandemic routines. Some felt that society’s harmful attitudes persisted despite the pandemic, which deepened their hopelessness and reluctance to pursue life goals, fearing sudden disruptions. Heightened emotional sensitivity led to panic attacks and frequent crying over minor disagreements.

*A42 (18 years old*,* female): I felt scared of not fitting in like everyone else seemed to. For example*,* with a group of friends*,* one would say something and the other would reply and they’d all laugh and everything*,* or they’d just know what to say*,* like in a joke conversation*,* one would say something and the other would know exactly how to answer. My fear was not knowing how to reply*,* for example*,* saying something and no one laughing at it*,* or things like that*,* just not fitting in.*

Others found shared spaces overwhelming and missed the solitude of lockdown, which they found more rewarding. As a result, some did not want to return to school, considering leaving or moving to virtual learning to avoid facing a world they no longer felt connected to.

*A42 (18 years old*,* female): At that time*,* I loved it*,* and in the end*,* when the news started saying that we have to go back to school*,* well*,* I racked my brains and actually begged my mum to enrol me in a virtual school*,* like studying online*,* but not have to be around other people again. So*,* the lockdown wasn’t so bad for me.*

### Interpersonal level

We now move to the interpersonal level of adolescent mental health, where we explore the relationships that adolescents maintain with others in their immediate social environment. Drawing on the the Critical Ecological Model of Adolescents’ Mental Health, this section highlights how connections with family members, peers, and romantic partners influence the way adolescents perceive and navigate their mental health. These relationships, as described by participants, play a central role in shaping adolescents’ emotional experiences, sense of support, and exposure to relational risks or challenges.

#### Positive, high-quality relationships

Emotional support from parents, caregivers, and extended family was identified as central to adolescents’ well-being. Trusted adults provided guidance, helped regulate emotions through open communication, validation, and unconditional support, even when mistakes were made. Grandmothers were especially significant, offering stability, advice, and comfort. Close relationships with cousins, aunts, and uncles also provided emotional and, at times, financial support, reinforcing adolescents’ autonomy and self-worth.

*A37 (17 years old*,* female): Sometimes I see other families that have an ugly stability*,* that is*,* dysfunctional families. But mine is not like that*,* mine is the complete opposite*,* you know? So sometimes when my mum sleeps in my bed*,* she tells me*,* “I love you*,* darling*,*” and I just think*,* “My God*,* I really do feel good with what I have”.*

High-quality family relationships were reported to have strengthened during the COVID-19 lockdown. The prolonged cohabitation fostered trust, mutual support, and dialogue. Intergenerational exchanges, such as sharing life stories or learning domestic skills like cooking, were valued. Ties with family members like cousins, who were often the only available peers, deepend, promoting solidarity. Collective coping strategies, such as reorganising households to care for infected family members, also enhanced a sense of unity during confinement.

*A11 (15 years old*,* male): So when I called my dad [separated parents/divorced]*,* I heard he was really unwell*,* I mean*,* really bad. So I told my mum*,* “Mum*,* my dad’s sick and he’s there all alone.” And she said […] “You know what? We should bring him to our house.” So my mum brought him to our place*,* she put him in my room*,* and since I had nothing to do all day*,* we weren’t in school*,* just doing virtual classes*,* I was the one in charge of making his home remedies.*

Supportive friendships were described as vital sources of calm, validation, and emotional security, particularly for adolescents facing family conflict or domestic violence. Bonds based on loyalty, shared interests, and authenticity provided crucial refuge from daily distress. Similarly, romantic relationships offered emotional support and stability, encouraging positive thoughts and helping avoid harmful situations, including suicidal behaviours or escalating family tensions. During lockdown, friendships and romantic ties that endured were sustained through persistence, empathy, and creative communication efforts, such as constantly messaging, video calls beyond academic matters, online gaming, and exchanging gifts via delivery apps. Once restrictions eased, adolescents valued reconnecting in person, despite lingering fears of contagion.

*A9 (17 years old*,* female): When I go out with them [my friends]*,* believe me*,* I feel like I’m in a completely different environment than at home*,* because they’re just very different from the situation I’m living in at home. So*,* I feel good in that moment*,* and then when I go back home*,* it’s like all that nice time I’d just spent is gone*,* because I return to my normal situation.*

#### Giving social support

Recognising others’ emotions and expressing kindness, compassion, and love were described as key relational values within adolescents’ accounts. Many expressed a desire to be perceived as trustworthy and empathetic, particularly towards family members, friends, or romantic partners. A recurring theme was their deep appreciation for the physical, emotional, and financial efforts made by their mothers, or in some cases, grandmothers, during their upbringing. Participants reported feeling a strong sense of responsibility to reciprocate this care by supporting their mothers emotionally, contributing to household tasks such as cooking and cleaning, and aspiring to improve the family’s economic situation and provide for their loved ones in the future.

*A38 (male*,* 18 years old): There was a time when my mum lost her job […] one time she couldn’t pay a bill*,* and I said*,* well*,* it’s okay*,* I’ll sell my [video game] console*,* I wasn’t really using it anyway. And I told her*,* like*,* it’s fine*,* I’ll sell it*,* and she was like*,* “No*,* no*,* how are we going to get out of this?” and so on… But I told her*,* it’s alright*,* we’ll sort it out later. So I sold it and paid the bill.*

In light of the emotional, social, and economic challenges experienced during the pandemic, adolescents valued the opportunity to support their families and peers in various ways. Additionally, they expressed pride when their parents were able to offer help to extended family, neighbours, or friends by lending money or providing food to those facing greater hardship.

*A8 (16 years old*,* female): Once*,* for example*,* a friend told me he didn’t have anything to eat for lunch*,* so I told my dad*,* and well*,* we weren’t doing that well ourselves either*,* but my dad told me to invite him over to have lunch*,* at least so he has something warm and decent for that day. So he came*,* had lunch*,* thanked us; we also gave him*,* I think*,* 30*,*000 pesos [7 $ aprox.] so he could buy some more food*,* and we gave him some rice and beans too.*

#### Loneliness and social isolation

Dissatisfaction with the quantity and quality of family and peer relationships was expressed by several participants, contributing to distressing experiences of loneliness, not only during the pandemic, but also as part of their broader social lives. They reported feeling isolated due to insufficient or low-quality time with parents, despite a desire for greater caregiver involvement. Loneliness was also associated with challenges in integrating into peer groups or lacking peers with whom to form emotional bonds. Additionally, negative experiences of loneliness were linked to significant losses, including the death of a close relative, family conflict, breakups, the end of friendships, or the loss of a pet.

*A34 (16 years old*,* male)*: *Let’s say that for a child*,* motivation doesn’t really come from friends or girlfriends*,* it comes from the family. Maybe people should pay more attention to young people*,* because sometimes we feel really alone when we don’t have family around*,* because that’s the most important thing you should have throughout your life… family. And well*,* in my case*,* I don’t have my family*,* they’ve never even asked about me. And that’s what makes me feel sad sometimes*,* because I don’t have a family. It’s as if I were dead.*

During the pandemic, many adolescents experienced profound loneliness despite sharing physical space with family members. They often felt disconnected from those they lived with, finding refuge in mobile phones, games, or social media. For some, parental job obligations left them alone at home for extended periods, while the lack of face-to-face peer interaction intensified feelings of isolation and sadness. The separation from grandmothers or extended family members, who were important sources of support, was a significant source of distress. Remote communication became the primary means of maintaining peer relationships, but limited internet access, superficial interactions, and frequent relocations disrupted friendships and romantic bonds. Adolescents who had recently changed schools or lacked established networks felt particularly isolated, and many reported frustration, sadness, and the challenge of rebuilding social ties post-lockdown.

*A6 (16 years old*,* male): Later on*,* you start to realise that sometimes you feel lonely*,* because even if you’re with your family*,* it’s not like you really share much with them. Well*,* back then [during the pandemic lockdown] I didn’t really spend much time with my family*,* I was basically always on my own*,* just on my phone.*

The loss of access to meaningful activities and social spaces during the pandemic was associated with feelings of monotony, boredom, and a diminished sense of youth. The cancellation of important events, such as fifteenth birthday celebrations (often accounted as culturally significant rites of passage for women in many Latin American countries), added to their disappointment. Time was perceived as dragging, routines became annoying, and many felt disoriented both physically and socially when restrictions eased.

*A2 (16 years old*,* female): It was like I couldn’t see them [friends]*,* or things like that*,* so we started to stop talking*,* we kind of began to lose that friendship*,* you know? I mean*,* we were together all the time at school*,* but chatting wasn’t the same*,* so we’d talk maybe once a month or so*,* and it just faded away…*.

#### Conflict-mediated family relationships

Due to inconsistent or hostile family environments, including financial disputes, parenting disagreements, infidelity, or separation, adolescents reported heightened anxiety, worry, and emotional instability. Such settings often prompted them to seek refuge with peers and distance themselves from family, leading to poor communication with parents. Many adolescents in patchwork families described difficulties adapting to and trusting a parent’s new partner, particularly when conflicts or domestic violence were present, which in some cases led them to move in with a grandparent or the other parent. Conflicts with siblings, half-siblings, or step-siblings, often stemming from personality clashes or perceived inequalities, further contributed to distancing from the home environment. Occasionally, disputes with other relatives, such as uncles, cousins, or in-laws, were also reported to negatively affect adolescents’ emotional well-being.

*A28 (15 years old*,* female): One moment he [brother-in-law] is fine with you*,* and then he gets angry because you don’t do him favours and things like that*,* even though he usually does nothing around the house*,* he just sleeps all day. […]*,* when he tried to cheat on my sister […] I’ve tried to talk to my sister about it*,* but […] she doesn’t believe me. Because of that*,* she stopped talking to me for about five months.*

Within the family environment, experiences of emotional neglect, physical violence, and psychological abuse were frequently described. A common concern was the absence of emotional validation, with participants describing their emotions and thoughts to be ignored, minimised, or misinterpreted. Physical violence involved hitting, slapping, kicking, or the use of objects, while psychological abuse included insults, humiliation, isolation, and threats, often linked to unmet parental expectations regarding academic performance, work, or personal life aspects, including sexual orientation. These experiences had significant impact on emotional well-being and self-esteem, with verbal abuse leaving lasting psychological harm. Excessive alcohol use by parents or grandparents was also reported, contributing to financial difficulties, domestic violence, and weakened emotional bonds due to adults’ frequent absence from home.

*A11 (15 years old*,* male): And then more and more problems*,* and there’s this thing when my mum gets angry*,* she says a word that destroys me*,* but she doesn’t even realise it*,* she focuses on saying that I’m useless*,* that I’m good for nothing. And that word*,* “useless*,*” I feel is really destructive.*

Exposure to domestic violence directed at their mothers by fathers or stepfathers was reported to affect the adolescents’ emotions and behaviour. Many witnessed physical and verbal aggression, leading to feelings of disappointment, anger, sadness, and insecurity. Some acknowledged replicating violent behaviours in school, while others felt compelled to protect their mothers, either by intervening during conflicts or urging them to leave the abusive relationship, despite potential economic hardship. In several cases, adolescents experienced direct abuse or chose to leave home temporarily. When mothers sought refuge by relocating to new cities or neighbourhoods, often with relatives or friends, adolescents faced the challenge of adapting to unfamiliar schools and social settings while supporting their mothers through the emotional and socioeconomic difficulties of starting over.

*A12 (16 years old*,* female): When I was about*,* what*,* five or six years old*,* he [father] was a very aggressive man*,* he used to hit my mum*,* he hit me too*,* he came home drunk*,* he was incredibly unfaithful to my mum. My dad abandoned us*,* and the worst part was that he took everything*,* everything*,* leaving us sleeping on planks and cardboard.*

Linked to conflicted marital relationships marked by domestic and gender-based violence, one parent often was absent in the adolescents’ lives. This absence disrupted affectionate and recreational bonds with fathers, leaving mothers, sometimes supported by grandmothers or aunts, to bear sole caregiving responsibilities. In some cases, fathers communicated manipulatively to gain information about the mother, generating feelings of threat and insecurity and prompting adolescents to avoid contact. For many, the circumstances surrounding parental separation were traumatic, involving exposure to violence and severe economic hardship that led to rejection of the father. Others described weak ties with fathers who showed disinterest, prioritised new families, misused alcohol, neglected financial responsibilities, and played little role in child-rearing, resulting in further distancing.

*A37 (17 years old*,* female): I mean*,* it’s complicated because if my mum doesn’t pressure him*,* he doesn’t give us anything*,* you know? And my mum doesn’t really push him*,* in the sense of saying: “Hey*,* remember you have children*,* you’ve got two*,* I can’t do everything on my own.” Even though she doesn’t earn much*,* she’s the one who provides for the household.*

Due to differences in opinions or emotional expression, adolescents reported tensions with their mothers. Recurrent arguments were linked to contrasting personalities and parenting approaches. Conflicts also arose when mothers disapproved of personal decisions, such as disclosing a diverse sexual orientation or initiating romantic relationships. Breaches of trust, such as sharing private information with extended family, led to emotional distancing. In one case, a mother projected resentment towards the father onto her daughter following a separation, deepening the adolescent’s feelings of sadness and rejection.

*A42 (18 years old*,* female)*: *She [my mum] argues with me a lot because she says I’m just like my dad and his family. […] Sometimes she says “You’re rubbish*,* just like your dad. You… you hate me*,* you’re going to leave me out on the street…” even though it’s not true*,* I really do support her a lot when it comes to my dad.*

During the COVID-19 lockdown, limited space and prolonged confinement led to frequent disagreements escalating into shouting and fights, causing adolescents’ frustration, anxiety, and distress, sometimes requiring hospitalisation. Some decided to live with relatives. Financial stress heightened caregivers’ irritability, lowering tolerance for disputes. Conflicts over shared spaces and resources increased among siblings, reducing adolescents’ autonomy and sometimes resulting in verbal or physical altercations. Adolescents with separated parents also struggled with limited contact due to infection fears, missing their usual time with their fathers.

*A28 (15 years old*,* female): We all started having clashes because we weren’t used to seeing each other that much […] So*,* there were several arguments when we began spending more time together. And not being able to go out or do many things made it worse.*

#### Challenges and risks in peer relationships

Difficulties in forming and maintaining peer relationships were commonly reported, with many participants describing challenges in expressing emotions or seeking help when feeling overwhelmed. These challenges sometimes fostered feelings of anger, resentment, and social isolation within school groups, increasing the risk of emotional regulation difficulties and limiting the development of supportive environments essential for their well-being. Aggression was a common response to interpersonal conflict; in hypothetical scenarios, many anticipated resorting to violence, indicating a tendency to rely on aggression as a primary strategy for managing conflict. Additionally, participants described engaging in verbal insults, physical aggression (such as hitting, kicking, or damaging objects), and prearranged fights.

*Observation note (30.03.2023): Some teachers are talking about two girls who arranged to meet at the park to fight. Not only the two girls showed up*,* but also their friends. They started hitting each other; one girl left the other with a facial injury*,* and in response*,* she received such a strong blow that it fractured her arm. She is currently in the hospital.*

Observations revealed that alcohol consumption frequently mediated peer interactions, often with the implicit or explicit consent of caregivers. Adolescents described parties where excessive drinking occurred, discussing its effects on behaviour. In some cases, students reported consuming alcohol within school premises, viewing it as a daring act without fully recognising any risks. School staff also noted the use of other psychoactive substances, such as marijuana, which adversely affected academic performance and overall well-being. Some adolescents reflected on these behaviours, acknowledging their potential to hinder personal goals and expressing interest in tools to support healthier choices.

*Observation note (17.03.2023): A group of friends brought aguardiente (an alcoholic beverage) into the school. Some students ended up noticeably drunk*,* prompting the teachers to realise what was happening and call the parents to inform them. […] In a meeting between one of the students involved*,* the father confronted the teenager*,* asking why he had to get himself into trouble if he knew that at home*,* whenever there’s alcohol*,* they’ve already shared some with him. He added that at home*,* they’ve drunk together openly several times*,* so why would he go and create problems at school?*

Romantic relationships were another important dimension of peer interaction. Adolescents involved in relationships marked by poor communication, lack of trust, or emotional support reported difficulties in emotional regulation, including anxiety, social withdrawal, and irritability. Breakups were often experienced as emotionally overwhelming, eliciting intense sadness, anxiety, and interpersonal conflict. Adolescents highlighted the importance of empathetic support from caregivers and peers, especially when experiencing feelings of rejection or low self-worth. In more severe cases, professional support was needed. Some used social media as an emotional outlet, while others exhibited impulsive behaviours such as cutting their hair.

*A42 (18 years old*,* female): This past December I had a boyfriend*,* and I gave him everything*,* I told my family about him and everything*,* and then*,* on December 24th at nine at night*,* he asked for a break. He said I was insecure*,* that I didn’t love him*,* and that I had made him feel really bad*,* and that was it*,* he let me go*,* I think that’s why I fell into depression and got anxiety.*

Peer relationships among adolescents often involve expressions of sexuality, including physical affection observed in school settings and events. Some accounts revealed risky sexual behaviours, such as unprotected group sex under the influence of alcohol or lack of knowledge about contraception and prevention of sexually transmitted diseases, underscoring the need for improved sexual education. The prominent position of sexuality among adolescents became clear even in public, and originally innocent behaviours were strongly sexualised.

*Observation note (31.10.2022): During the Halloween celebration*,* students from different grades organised an improvised party in one of the school’s classrooms*,* where dancing became the central form of interaction. They wore revealing costumes*,* in line with current youth trends*,* and danced in pairs or trios using sensual body movements*,* typical of ‘perreo’ and other popular urban dacing styles and cultural trends. Physical closeness*,* bodily contact*,* and expressive movement were frequently observed among peers during interactions*,* particularly in shared activities and dance*,* reflecting patterns of social engagement.*

The pandemic intensified challenges in maintaining social connections, as adolescents increasingly relied on virtual means of interaction. Mobile phones became their primary resource, often used for streaming and social media. Many reported neglecting responsibilities and experiencing feelings of purposelessness, worsened by negative social comparisons and reduced family interaction. Teachers and counsellors expressed concern about behaviours observed during and after lockdown, including the sale of sexual images via online platforms, exposure to suicide-promoting content, and excessive pornography use.

*C1 (female*,* psychologist): So*,* another important thing is that here at school*,* the issue of child pornography became evident*,* for example*,* or boys who started watching platforms where they could sell sexual content […] well*,* there were probably boys who thought: “Well*,* here I can make some money.” However*,* there was also social pressure.*

The data also revealed frequent engagement in sexting, reported exclusively by female participants. Most met their partners online, via social media or video games, and were later asked to share explicit content. Although they initially consented, many experienced distress due to fears about potential consequences, such as physical punishment and/or confiscation of phones by their parents. Despite this, the adolescents resumed their behaviour once devices were returned. One participant had her images publicly shared, resulting in bullying and a school transfer. Several interviewees reported failed attempts to video-call supposed foreign partners, particularly during the COVID-19 lockdown.

*C5 (female*,* psychologist): Now*,* another thing that I think also increased during the pandemic was that we don’t see each other*,* we don’t have that contact*,* so we use the phone. And there’s something that*,* I don’t know if it’s a coincidence*,* but right now it’s completely out of control*,* the misuse of social media in terms of sexting*,* grooming*,* and all these situations where you see adolescents acting like it’s completely normal without thinking about the risks involved.*

Prolonged hours spent gaming online, often with peers or strangers, led to patterns of dependency in several instances. Although gaming served as a form of socialisation during isolation, it often interfered with school and home responsibilities, and relationships formed through gaming were viewed as potentially unsafe by parents. Some of the adolescents mentioned gaming as a form of escape. One participant even reported practising “reality shifting,” a technique learned online to escape to an idealised alternate reality mentally. She described following specific routines and participating in social media groups dedicated to this phenomenon.

*A10 (14 years old*,* female): I became really obsessed*,* and that’s why I couldn’t manage it*,* because I obsessed over changing and wasn’t satisfied with this reality. I kept asking*,* “Why do I exist here? Why can’t I exist somewhere else?” But this year I found out that you can’t do that*,* because you’re putting pressure on your reality and your consciousness feels pressured and doesn’t travel […] Why? For fun*,* because I want to experiment to see if it’s real and with that you can mentally know many things.*

#### Sexual violence

Sexual violence was present in various forms across participants’ accounts. Reports included a male adolescent who had faced legal proceedings for alleged sexual assault, later dismissed, and another who disclosed being abused at age six by a family friend. A female participant described ongoing sexual harassment and an attempted assault by a male cousin, leading her to adopt constant protective measures while continuing to live with him; disclosures to adults were met with inaction. One girl shared that her first sexual encounter occurred at the age of eight, constituting sexual violence under Colombian law. School counsellors observed a rise in sexual abuse cases during the COVID-19 lockdown, linked to prolonged proximity to perpetrators within the home and reduced access to external support, with many cases only surfacing after students returned to school.

*A20 (14 years old*,* female): I was just hangig out in my room*,* and he came in whileI was getting dressed and tried to force himself on me […] my cousin was there*,* she was the one who came in and helped me […] so my family took measures*,* like keeping a safe distance; well*,* social distancing and all that. And my mom said no*,* now she’s more careful with me.*

### Community level

At the community level, we examine how adolescents’ mental health is shaped by the broader social connections in the communities in which they live. Guided by the the Critical Ecological Model of Adolescents’ Mental Health, we explore the collective dynamics, shared experiences, networks of support, as well as local risks or challenges that influence how adolescents feel, act, and seek socioemotional help within their environments. Although fewer data addressed community-level factors compared to the personal, interpersonal, and organisational levels, these accounts were analysed in depth and retained as a distinct analytic level, reflecting a coherent and theoretically meaningful pattern without extending beyond what the empirical material could robustly support [29].

#### Groups and solidarity

Accounts highlighted involvement in local foundations that created safe and supportive environments for social interaction outside of school. They also referred to youth groups within church communities, as well as participation in extracurricular activities such as sports, dance, and theatre. In response to economic hardship during the COVID-19 lockdown, local communities developed their own support strategies, such as organising food and donation drives, known as *donatones* or “solidarity teams”, to assist those in urgent need. In Bogotá, hanging a red cloth in the window became a widely recognised symbol of food insecurity. Some adolescents reported participating in these collective efforts.

*A8 (16 years old*,* female): Sometimes there were people who asked for some groceries*,* they organised “solidarity teams” in the administration for those most in need in the housing complex. They registered apartments that said*,* “No*,* sorry*,* I need milk*,*” “I need eggs*,*” so sometimes donations were made; they took rice*,* beans*,* for those people.*

#### Disadvantages and violence

Neighbourhoods where the interviewed adolescents lived lacked organised spaces, with narrow, poorly maintained access roads and unfinished buildings. Some areas emitted unpleasant odours from neglected natural water sources. While commercial establishments, such as markets and workshops, were present, certain hidden zones included recycling depots, car and motorcycle garages occupying public space, and sex work venues. Various forms of pollution, air, noise, visual, and litter, were evident. Insecurity was also a concern of participants, with reports of high crime rates, including drug dealing, street fights, threats among urban groups, and armed robberies.

*A30 (12 years old*,* female): Next to my house they sell drugs*,* like marijuana and stuff. And*,* uh! Sometimes it really annoys me.*

### Level of organisations and mass media

At this level, we explore how formal institutions (such as schools, healthcare systems, and religious organisations) and mass communication platforms (including social and digital media) shape adolescents’ mental health experiences. Framed by the the Critical Ecological Model of Adolescents’ Mental Health, we consider how these structures influence access to support, shape norms and expectations, and mediate adolescents’ understanding of well-being.

#### School

During the COVID-19 lockdown, many adolescents faced significant barriers to learning, including a lack of access to computers or stable internet connections, often relying on shared mobile devices. Inadequate study environments, such as following lessons from bed, further undermined effective engagement. Students reported low comprehension during virtual classes, frequent connectivity problems, and an overwhelming workload due to self-directed study materials, while the absence of peer interaction limited both collaborative learning and emotional support. These challenges led to low motivation, disengagement, and decline of academic achievement, with some adolescents failing the school year or describing it as a complete loss of learning. Schools attempted to mitigate the impact by offering virtual mental health workshops and maintaining contact with students and parents.

*A37 (17 years old*,* female): Me without internet*,* I remember I didn’t even have a phone either. My mum let me use hers*,* but since it was her work phone*,* she wouldn’t really let me have it. My sister lent me a computer*,* but only for half a day because she needed it. So during the pandemic*,* I really fell behind*,* a lot*,* not just a little*,* I mean*,* my schoolwork was very poor.*

Participants benefitted from meals at school before and after the lockdown which served as essential economic support for low-income families. During the pandemic, this support was given in the form of food vouchers, helping families to cope with increased hardship and thus, promoting the adolescents’ well-being and mental health.

*A37 (17 years old*,* female): I’m really grateful to the school because during the pandemic*,* they gave vouchers to children from all schools. My brother got 50*,*000 [12 $ aprox.] and I got 50*,*000 […] it would come in an email saying where to pick it up*,* like at the “name of the store*,*” and 100*,*000 two years ago*,* that was a lot*,* I mean*,* it really was quite a bit […] I’m very thankful because honestly*,* it helped us through a lot.*

#### Mental health promotion and care

Many adolescents recognised the value of professional psychological support and expressed a desire to consult with a psychologist. However, these requests were not always well received by parents, either due to stigma or financial constraints. Those who sought care through the health system frequently encountered barriers such as long waiting times, limited session durations, and a lack of continuity. These limitations led to dissatisfaction and disillusionment, often resulting in treatment discontinuation and reliance on family beliefs, home remedies, or self-medication instead.

*A4 (female*,* 15 years old): I think I suffer from anxiety […] my grandma told me to take this thing*,* it’s like a pill*,* I don’t know what it’s called*,* but she gives it to me and it calms me down… Or she gives me very cold water with ice*,* really cold*,* and that helps too.*

The internet provided the most readily available mental health support for several participants, particularly in the absence of offline alternatives. They often turned to self-help information available on the internet or follow social media accounts that shared content related to mental health and emotional well-being.

*A8 (16 years old*,* female): I used to spend a lot of time watching videos about self-love and how to improve yourself. Out of curiosity*,* I started watching to see what it was about. At that time*,* I wasn’t feeling bad exactly*,* but I didn’t feel great either. It was more like I was just existing […] Those videos taught me it’s okay to feel*,* it’s okay to have self-love*,* and it’s okay sometimes to feel low because that’s just part of who you are.*

While not a primary or consistent source of mental health support, religious institutions such as the church were found as secondary spaces for some families during the pandemic. Some participants mentioned that religious practices became more private during the lockdown, leading to a temporary disengagement. However, in certain cases, regular attendance resumed once restrictions were lifted, suggesting that, for some families, religious spaces continued to play a supplementary role in providing emotional or spiritual support. Although less prominent than schools or digital media, these institutions remained part of the broader landscape through which adolescents might navigate emotional challenges.

*A19 (12 years old*,* male): Well*,* on Saturday… I mean*,* we go to church because on Saturdays they have activities for kids and at… at… 4:00 in the afternoon they have activities for young people.*

## Discussion

This study explored the mental health of adolescents living in urban contexts characterised by social inequality, including the benefits and challenges associated with the COVID-19 lockdown. Data were collected through semi-structured interviews and direct observations with adolescents and school counsellors in two disadvantaged neighbourhoods in Bogotá, Colombia. Using reflexive thematic analysis, themes were first generated inductively from participants’ accounts and later organised deductively through engagement with theoretical concepts. On this basis, we developed the Critical Ecological Model of Adolescents’ Mental Health, which presents four interconnected levels: (a) personal, (b) interpersonal, (c) community, and (d) organisations and mass media. This model is based on the previously established Mental Health and Well-being Ecological Model [21].

Adolescents demonstrated multiple capacities to support their mental health, including engaging in self-care and developing life plans aimed not only at personal growth but also at improving family conditions. These behaviours align with evidence showing that adolescent self-care includes daily actions to promote health, regulate emotions, and seek support when needed, while life projects are influenced not only by individual aspirations but also by structural inequalities that shape opportunities for social mobility [43–45]. Our results mirror previous research showing that supportive family environments, characterised by cohesion, open communication, and low conflict, enhanced resilience and emotional competence, while adult guidance helped adolescents interpret and regulate emotions [10, 11, 46]. Reciprocity and empathy emerged as key social skills, enabling trust, negotiation, and positive peer interactions [45, 47]. In contexts where familial support was limited, peers often provided compensatory emotional safety and intimacy. The significance of peer relationships during adolescence has been well documented with research indicating that friendships and romantic relationships are central to social development and identity formation, depending on trust, emotional competence, and conflict management skills [48–51].

Despite these strengths, adolescents faced significant mental health challenges. Emotional distress, depressive symptoms, low self-esteem, and in some cases self-injury or suicidal behaviour were reported. Our findings extend earlier evidence by showing that many difficulties originate in conflictual family environments, including sibling disputes, strained parent–child dynamics, and maltreatment, fostering dissatisfaction, mistrust, and emotional dysregulation [52–55]. In our study, adolescents reported high levels of intrafamilial violence, often exacerbated by caregivers’ problematic alcohol use. These patterns, rooted in a cultural context where physical punishment and alcohol consumption are normalised, produced significant harms such as anxiety, depression, substance use, suicidal behaviour, and the perpetuation of violence across generations [54, 56–60]. This study highlights that gender-based violence against mothers added further stress, with adolescents reporting hypervigilance, insecurity, and distress, consistent with evidence linking such experiences to diminished life satisfaction and perpetuation of aggressive behaviours [61–63]. Additional challenges included bereavement, academic pressures, and dissatisfaction with self-image, all contributing to feelings of frustration, hopelessness, and social isolation, echoing evidence from previous research [64–67].

Exposure to sexual violence within adolescents’ own families was also reported, often involving individuals they lived with, particularly during periods of prolonged confinement, such as the COVID-19 lockdown. Extended proximity to potential perpetrators, coupled with limited access to external protective environments, intensified adolescents’ vulnerability to abuse [68, 69]. Some participants described that disclosure of abuse was minimised or ignored by family members, deepening feelings of insecurity and isolation, while persistent unsafe living arrangements reflected systemic failures to provide timely and effective interventions. These findings emphasise the urgent need to strengthen child protection mechanisms, raise community awareness, and ensure access to psychosocial support services, particularly in socially vulnerable contexts [70].

Adolescents also encountered age-specific challenges associated with identity formation and independence. Peer relationships were sometimes shaped by exposure to psychoactive substances, particularly alcohol, which was easily accessible and rarely restricted. Such patterns mirror findings from previous research, which links adolescent alcohol consumption to low family functioning, ease of access, and permissive parental attitudes [71, 72]. Romantic relationships, while central to social development, could be intense and destabilising, with conflicts or breakups contributing to loneliness and emotional instability [73]. Sexual activity often occurred with limited adult guidance, leading adolescents to rely on peers or online sources, where misinformation was common. Previous research similarly points to contextual factors such as violence, substance use, school dropout, and asymmetric relationships where male partners exert control, which further undermines adolescents’ autonomy and safety in navigating sexuality [74–76]. Observations of highly sensual dance styles at school events illustrate how adolescents also express their sexuality performatively, reflecting peer norms, cultural influences, and popular media rather than inherently inappropriate behaviour [77]. A more concerning practice identified was sexting, which reflects unequal digital dynamics that can expose adolescents to reputational harm, privacy violations, grooming, and exploitation [78, 79]. This study supports prior work showing that digital technologies provided connection but also fostered harmful self-comparisons, body dissatisfaction, cyberbullying, and in severe cases suicidal behaviour [78, 80]. Excessive video gaming was associated with anxiety, musculoskeletal strain, and problematic behaviours including substance use and gambling [78, 80]. A finding that was surprising to us in this study was the practice of “reality shifting” during the pandemic, which appeared to function as a coping strategy; although non-pathological, it raises questions about long-term effects on adolescents’ agency and sense of control [81].

The COVID-19 pandemic acted as an amplifier of adolescents’ mental health outcomes, deepening historically rooted social inequalities and accentuating both benefits and challenges. Rather than creating new vulnerabilities, the lockdown exacerbated processes of long-standing social and economic precarisation that already shaped adolescents’ everyday lives, undermining adolescent well-being by disrupting daily life and intensifying stressors, with vulnerable groups such as low-income families, migrants, and those in overcrowded housing being disproportionately affected [25, 82, 83]. Bereavement, particularly following the loss of close relatives, was a major challenge, with adolescence recognised as a stage requiring tailored support to prevent isolation and complications [84]. Our findings are consistent with previous research indicating that for those adolescents with healthy family dynamics prior to the lockdown, the additional time spent together raised greater cohesion, improved communication, and enhanced empathy as well as fostered self-esteem and self-image, ultimately strengthening resilience and emotional competence [11, 46, 60]. Conversely, in families already facing structural disadvantage, the prolonged confinement exposed and intensified underlying conflicts linked to material deprivation, limited living space, and forms of violence that had previously remained latent, resulting in heightened distress and insecurity [23, 85, 86]. These contrasting experiences illustrate how supportive environments can buffer the effects of crisis, while historically produced conditions of precarisation exacerbate emotional distress, underscoring the role of structural inequalities in shaping adolescent well-being during times of disruption.

Fieldwork for this study was conducted during the period when adolescents were returning to in-person schooling after approximately 18 months of lockdown. This timing offered real-time insights into their coping processes in response to the transition. Participants employed a range of coping strategies, which could be categorised as problem-oriented, disruptive, or avoidant [87, 88]. Previous research shows that ineffective coping is associated with poorer mental health outcomes, particularly following the disruptions and containment measures related to the COVID-19 pandemic [87, 88]. These findings underscore the importance of evaluating the effectiveness of adolescents’ coping strategies and strengthening those that are more adaptive in the educational setting [88].

Our study also highlights that emotional distress and mental health capacities are not isolated or purely individual phenomena, but rather the outcome of historical, social, and structural processes expressed in everyday life [15, 27]. Adolescents’ lives are shaped by a capitalist and patriarchal system that undermines their well-being and reproduces social inequality [14–16]. Within this context, the feminisation of care emerges as a key structural process through which gendered inequalities are reproduced in daily family life. Single-parent households headed by women exemplify how caregiving responsibilities are disproportionately borne by women, placing mothers under significant economic and emotional strain. This concentration of unpaid care labour intensified maternal stress and intrafamilial conflict, while simultaneously increasing adolescents’ exposure to emotional burden and reducing the family’s capacity to function as a protective environment [89, 90]. Economic hardship, limited employment opportunities, and a lack of social security constrained families’ living conditions. This study also reported neighbourhoods as unsafe, violent, and polluted, where exposure to insecurity and multiple forms of violence constituted a normalised feature of everyday life rather than an exceptional occurrence, limiting opportunities for recreation and the development of social support networks. This normalisation of violence, manifested across family, community, and public spaces, shaped adolescents’ emotional worlds by fostering chronic states of fear, hypervigilance, and insecurity. Social programmes such as school feeding schemes played a crucial role in meeting basic needs while also reflecting the fragility of structural support for well-being in contexts where violence is persistently managed through individual and familial coping rather than collective or institutional protection.

Within this broader context of accumulated structural disadvantage, access to mental health care is further constrained by systemic limitations that disproportionately affect adolescents living in conditions of social inequality. In Latin America, and particularly in Colombia, mental health care remains underfunded and institutionally fragmented, resulting in a substantial treatment gap despite relatively high overall health service utilisation [91, 92]. Barriers such as long waiting times, limited availability of specialised child and adolescent services, and the concentration of trained professionals in urban centres restrict timely and adequate care for young people [91, 93–95]. These limitations are compounded by the predominance of a biomedical model and persistent stigma, which marginalise psychosocial approaches and place the burden of coping on families and individuals [91, 92]. Consequently, structural weaknesses in the mental health care system operate as macro-level determinants that exacerbate adolescent psychological suffering in highly unequal urban contexts. Importantly, these structural constraints are not unique to the Colombian context. Qualitative studies conducted in other highly unequal Latin American urban settings that experienced prolonged lockdowns, such as Buenos Aires, urban areas of Chile, and the periphery of Mexico City, similarly document how the pandemic intensified long-standing processes of socioeconomic precarisation, housing overcrowding, educational and digital inequalities, and the disruption of adolescent sociability, contributing to heightened emotional distress, anxiety, and depressive symptoms [23, 86, 96, 97].

Based on these findings, the Critical Ecological Model of Adolescents’ Mental Health was developed as a conceptual synthesis that articulates the interconnected levels of personal and family relationships, community dynamics, institutional interactions, and the pervasive influence of mass media and the internet. The model illustrates how structural inequalities become embodied in adolescents’ everyday experiences, shaping family, community, and institutional relationships, while also revealing the emergence of capacities for care, solidarity, and agency within contexts of adversity [15, 21]. Mental health is thus understood not as an isolated or individual phenomenon, but as the outcome of social and economic processes operating across all levels of adolescents’ lives [15, 21]. Although the model adopts a multilevel structure commonly associated with ecological approaches, its contribution lies in a critical reworking of ecological thinking grounded in Latin American Social Medicine and the Social Determination of Health [15, 98]. Rather than following a multicausal or functionalist logic, the levels function as an analytical device through which structural inequality is conceptualised as a constitutive force permeating all dimensions of social life. Each level is therefore interpreted not as an independent ‘factor’, but as a situated expression of broader historical processes of social and economic precarisation that shape adolescents’ living conditions, subjectivities, and possibilities for action. In this sense, the model constitutes a contextualised adaptation of ecological frameworks for highly unequal urban settings in Latin America, where normalised violence, the feminisation of care labour, and the structural fragility of health systems are not peripheral influences but central determinants of adolescent mental health. By foregrounding these structural conditions, the model offers a substantive conceptual contribution to research and intervention in contexts where conventional ecological models may insufficiently capture the depth and persistence of social inequality.

### Strengths and limitations

The fieldwork conducted for this study provided adolescents with a safe and open space in which to speak about their socioeconomic conditions, mental health, and experiences during the COVID-19 lockdown. This approach made it possible to access deep and meaningful aspects of adolescent mental health, expressed in their own voices and through their own narratives. This way, the interviews, together with the observational records, offer a comprehensive view of the mental health situation of adolescents living in an urban context marked by social inequality.

The timing of the fieldwork constitutes a key strength rather than a limitation of this study. Data were collected during 2022–2023, precisely when adolescents were returning to in-person schooling after prolonged COVID-19 lockdowns. This moment enabled the collection of rich narratives from adolescents who were highly motivated to share their experiences, while memories of confinement, loss, and disruption remained recent. This temporal proximity provided valuable insights into the lived mental health consequences of an exceptional historical period.

Several limitations should nonetheless be acknowledged. First, the study included only adolescents enrolled in and accessible through the formal education system. As such, the perspectives of those who have dropped out of school or who do not attend regularly, groups that may face higher levels of social exclusion, are not represented in the data. This may limit the diversity of mental health experiences captured, particularly among the most marginalised urban youth. To address this, field observations were also conducted in neighbourhood spaces, which helped to broaden the scope of the data collected beyond the school environment.

Second, participation required written parental consent. Some adolescents expressed interest in the study but were unable to take part due to a lack of authorisation from their caregivers. This may have resulted in the exclusion of perspectives from adolescents living in more restrictive or conflictive family environments, contexts that are particularly relevant when exploring mental health under conditions of social inequality. However, the data collected through field observations and interviews with school counsellors helped to enrich and complement the information gathered directly from adolescent participants.

Third, neighbourhood observations were conducted as direct non-participant observations, in some cases from a vehicle for safety reasons. While this limited prolonged immersion in the neighbourhoods, the aim of these observations was contextual description rather than ethnographic engagement with residents’ daily lives. Nevertheless, this constraint is acknowledged as a methodological limitation.

Fourth, the sample reflected limited sexual and migratory diversity, as most adolescent interview participants were cisgender and predominantly heterosexual, which may limit the perspectives captured regarding mental health in gender- and sexuality-diverse adolescents. However, both interviews and observations explored experiences related to diverse identities, partially mitigating this limitation.

Finally, not all members of the research team were fluent in Spanish, the language in which the data were collected. This created reliance on translation and the interpretive work of bilingual researchers, with potential loss of nuance. At the same time, the process fostered reflexivity and collaborative dialogue, encouraging the team to question assumptions and make analytic decisions more transparent.

## Conclusion

Adolescents’ mental health in urban contexts of social inequality is shaped by structural, historical, and cultural factors that often contribute to sadness, anxiety, frustration, and, at times, depression. Using the Critical Ecological Model of Adolescents’ Mental Health, this study found that these challenges extended beyond the individual to family, peer, community, organisational, and media contexts. Yet adolescents were not passive recipients of adversity; they showed agency through self-care, life planning, empathetic relationships, community solidarity, and constructive engagement with organisations and mass media.

The COVID-19 pandemic, and particularly the lockdown, intensified both the risks and the protective factors associated with adolescent mental health. On the one hand, confinement exacerbated socioeconomic hardship, interpersonal conflict, and behavioural difficulties, deepening existing inequalities. On the other hand, it also created opportunities for the strengthening of resilience, with some adolescents reporting enhanced self-care practices, greater family cohesion, and stronger community bonds. These findings highlight the dual nature of the pandemic experience and reinforce the importance of addressing adolescent mental health within its broader social context.

Building on these findings, interventions to strengthen adolescent mental health and well-being should address multiple, overlapping social and structural inequalities, including gender and socioeconomic status, while operating across the levels of the Critical Ecological Model of Adolescents’ Mental Health. From an applied perspective, the model helps to identify concrete institutional entry points for intersectoral action, particularly involving social sector government agencies responsible for health, education, and child protection, and municipal governments, whose coordinated efforts are essential to move beyond fragmented responses.

At the personal level, programmes promoted jointly by the education and health sectors can support adolescents’ capacities for self-care and emotional regulation, particularly in urban settings where access to specialised mental health services is limited. At the interpersonal level, initiatives coordinated with child protection agencies and local social protection services may strengthen parent–adolescent relationships, family cohesion, and caregiving networks, especially in contexts marked by gender-based violence or female-headed households, by providing caregivers with emotional, social, and practical support. Peer-focused initiatives should offer safe spaces to build friendships and navigate risks, such as substance use or digital exposure, in ways that promote supportive, non-stigmatising relationships.

At the community level, local governments, in collaboration with community-based organisations, neighbourhood groups, and cultural or sports programmes play a key role in sustaining safe spaces for adolescents and expanding opportunities for well-being, such as access to artistic, recreational, educational, and vocational activities. Strengthening these initiatives requires not only targeted funding but also stable public policies aimed at improving the socio-economic conditions of urban neighbourhoods, recognising social inequality as a central determinant of mental health problems.

At the organisational level, schools emerge as key preventive spaces, not as substitutes for mental health care, but as settings for early identification of emotional distress, everyday psychosocial support, and referral to specialised services when needed. Consolidating schools as reliable support networks requires coordination with health and social services, the protection of programmes such as school feeding schemes, and the integration of educational and psychosocial approaches within school environments. In parallel, community-based mental health and psychosocial support services are needed in vulnerable neighbourhoods to complement the work of schools and the health care system, helping to reduce barriers to access, stigma, and service overload.

The different levels should not be viewed as merely external conditions, but as active forces that shape adolescents’ mental health. Therefore, effective responses must go beyond individual-level interventions and engage with the social, economic, and political roots of inequality. Promoting adolescent mental health in urban Latin American settings thus requires sustained intersectoral strategies, led by public institutions and grounded in community participation that seek not only to mitigate distress but to transform the structural conditions that undermine collective well-being.

## Supplementary Information

Supplementary Material 1.

Supplementary Material 2.

Supplementary Material 3.

Supplementary Material 4.

## Data Availability

The data collected and analysed in this study are not publicly accessible, in accordance with ethical protocols and confidentiality agreements. Participants did not provide consent for their information to be shared outside the research team, and as such, data availability is restricted. Requests for further information about the data or materials can be directed to the corresponding author [johanna-carolina.sanchez-castro@charite.de](mailto: johanna-carolina.sanchez-castro@charite.de) .
