# Supplementary material for: Adolescent mental health and social inequality in the aftermath of COVID-19 in Bogotá, Colombia: a qualitative study using a critical ecological model
Source: BMC Public Health. 2026 Jan 21;26:347. doi: 10.1186/s12889-026-26293-9 (PMC12849467; doi:10.1186/s12889-026-26293-9)
Supplement: Supplementary file 1 — Supplementary Material 1. [file 12889_2026_26293_MOESM1_ESM.pdf]

Additional file 1: Observation guide

Mental health and social inequality in the aftermath of the COVID-19 pandemic – a qualitative study among adolescents living in Bogotá, Colombia

Johanna Carolina Sánchez-Castro, Nelly Esther Caliz Romero, Laura Pilz González, Christiane Stock, Katherina Heinrichs.

This guide was designed to support the direct observation sessions conducted in schools and neighbourhoods. It includes the instructions for the researcher to record field notes related to adolescents' mental health, evidence of social inequality and challenges associated with the COVID-19 pandemic.

<u>Observation guide:</u>

The researcher must always keep the field diary readily available, ensuring that all observation activities are recorded in a timely and appropriate manner.

Notes should be kept in an organised manner, and their construction should consider the characteristics of the records in Table A.

Table A: Characteristics of the field notes

Characteristic	Definition
Description of the observed situation	This involves providing an objective account of the context in which the action takes place. It includes brief notes on the relationships and situations of the individuals within the observed setting and everyday life. Speech or conversations may be included, but no personal data should be recorded.
Impressions	These refer to the subjective record of the observations and are important because they help the researcher evaluate and make sense of what is being observed. They include introspective comments about what the researcher sees and experiences, as well as what these experiences mean to them. Therefore, impressions should be recorded for all observations.
Preliminary reflections	These refer to the interpretations that allow the researcher to examine the elements recorded in the two previous sections. They help to explain what has been observed and to highlight why the observations were relevant to the research. In this section, the researcher should begin analysing the elements emerging from the description, potentially using preliminary analytical categories.
Theoretical notes	These involve relating the described situations and interactions to relevant theoretical frameworks for further analysis. This section initiates a theoretical argument aimed at understanding how the observed elements function within the research problem or object of study.
