# Supplementary material for: Adolescent mental health and social inequality in the aftermath of COVID-19 in Bogotá, Colombia: a qualitative study using a critical ecological model
Source: BMC Public Health. 2026 Jan 21;26:347. doi: 10.1186/s12889-026-26293-9 (PMC12849467; doi:10.1186/s12889-026-26293-9)
Supplement: Supplementary file 3 — Supplementary Material 3. [file 12889_2026_26293_MOESM3_ESM.pdf]

Additional file 3: Problem-centred interview guide for counsellors

Mental health and social inequality in the aftermath of the COVID-19 pandemic – a qualitative study among adolescents living in Bogotá, Colombia

Johanna Carolina Sánchez-Castro, Nelly Esther Caliz Romero, Laura Pilz González, Christiane Stock, Katherina Heinrichs.

This problem-centred interview guide was developed to explore school counsellors' perspectives on adolescents' mental health in contexts of social inequality. The questions address counsellors' professional experience working with adolescents, counsellors' observations of the effects of social inequality and COVID-19 control measures on adolescents' lives and emotions, and perceptions of the relationship between social inequality and adolescent mental health. The guide is flexible, allowing the interviewer to provide prompts, definitions, and clarifications to ensure shared understanding, while encouraging open-ended and reflective responses from participants.

Introduction - presentation:

My name is Carolina Sánchez Castro, and as part of my doctoral thesis at the Institute of Health and Nursing Science, Charité – Universitätsmedizin Berlin (Germany), I would like to explore how social inequalities influence the mental health of adolescents in Bogotá D.C., particularly during the COVID-19 pandemic.

That is why I would like to invite you to use this space to share your thoughts, opinions, and experiences on the topic. Our conversation will last approximately 45 minutes in the form of an interview. I will also ask you some general questions about your gender and profession.

Please remember that this is a safe space and that there are no "right" or "wrong" answers. Everything you share with me will remain confidential. I'd also like to remind you that your participation is entirely voluntary, which means you can choose to stop answering or leave the interview at any point. If there is any question you'd rather not answer, just let me know, you are not obliged to respond.

Please remember that this interview will be recorded. I'd like to ask if you agree to this and if you allow me to use a voice recorder during our conversation. All your personal information will remain confidential, and I will make sure that none of your responses can be traced back to you. Also, don't be surprised if I take notes during our talk, this is just to help me remember important information and possibly come back to it later. Please don't let it distract you.

Do you have any questions so far?

All this information is also included in the informed consent form, which have signed. By signing that document, you confirmed your consent to participate and agreed to the processing of your personal data.

Demographic questions

1. Which is your gender?
2. Which is profession?

Start of the Interview

(Audio recording begins)

The following questions are related to the objectives of the research and will be asked during the interviews with the participants (Table D):

Table D: Questions for counsellor participants

Category	Questions
Ice-breaker question	1. Could you tell me about your work with adolescents?
Familiarity with social inequality	2. How familiar are you with the term *social inequality*?<br><br>*Follow-up: If the participant says they are not familiar with the term, a brief explanation will be provided to help them understand it and relate it to their own experiences or knowledge.*<br><br>Based on their answer, you may ask whether the participant knows the concept but refers to it by another name, or whether they have experienced situations in their life that are related to it and that would allow them to continue participating in the interview. The aim is to build a shared understanding of the term *social inequality*, so that both interviewer and participant are on the same page during the conversation.<br><br>Definition of social inequality:<br><br>The definition will be printed and made available so that the participant can read it easily.
Feelings related to social inequality	3. What feelings have you observed in adolescents as a result of social inequality?
Familiarity with COVID-19 control measures	4. What pandemic control measures have affected adolescents' lives – and how?<br><br>*Follow-up: If the participant says they are not familiar with the disease control measures, a brief explanation will be provided to help them become familiar with the topic.*<br><br>Examples of COVID-19 control measures:<br><br>*These will be printed and available for the participant to view if needed.*

Category	Questions
Influence of COVID-19 control measures and social inequality on adolescents' lives	5. What changes do you think adolescents have experienced as a result of the COVID-19 control measures?<br>6. In what ways do you think the social inequality experienced by adolescents has been influenced by COVID-19?
General aspects of mental health	7. Now I would like to talk about some aspects of adolescents' mental health. What aspects or situations do you think are relevant to adolescents' mental health?
Changes in adolescents' mental health related to social inequality and COVID-19 control measures	8. How do you think social inequality affects adolescents' mental health?
	9. How do you think adolescents feel about the social inequality they experience and the COVID-19 control measures they were subjected to?
	10. How do you think adolescents' life plans have been affected by social inequity, especially during the pandemic?
	11. Thank you for telling me about your work with adolescents. I would like to know, what do you think could help improve this situation?
Final	12. Is there anything else you would like to add?
