# Supplementary material for: Adolescent mental health and social inequality in the aftermath of COVID-19 in Bogotá, Colombia: a qualitative study using a critical ecological model
Source: BMC Public Health. 2026 Jan 21;26:347. doi: 10.1186/s12889-026-26293-9 (PMC12849467; doi:10.1186/s12889-026-26293-9)
Supplement: Supplementary file 4 — Supplementary Material 4. [file 12889_2026_26293_MOESM4_ESM.pdf]

Additional file 4: Original Spanish-language quotes

Mental health and social inequality in the aftermath of the COVID-19 pandemic – a qualitative study among adolescents living in Bogotá, Colombia

Johanna Carolina Sánchez-Castro, Nelly Esther Caliz Romero, Laura Pilz González, Christiane Stock, Katherina Heinrichs.

This supplementary file contains the verbatim Spanish-language quotations used to illustrate the findings of the study. Each quote corresponds to a translated excerpt presented in the main manuscript, allowing readers to verify meaning and nuance in the original language (Table E). The quotes are presented in the order in which they appear in the main article and listed using the same identification codes.

Table E: Translated and original quotes

Quote ID	English Version (as published)	Original Spanish Version
**A37**	Alone, alone, umm, well, it's not that I have a big ego, but I also used to say to myself, but I'm not ugly, I mean, I don't know, so what if that girl has a better body or because she's chubby or more whatever, you know? Umm, yeah, like, I started to recover that confidence on my own. So I would ask my mom, "Oh, buy me some makeup, then, ah, buy me this," and I started dressing nicely, feeling pretty, you know? Especially my face, because like, my body isn't something I love, but it doesn't bother me that much.	Sola, sola, ehhh yo bueno, no es que tenga el ego alto, pero como que yo también me decía, pero yo no soy fea, yo qué sé yo, porque esta muchacha tenga mejor cuerpo o porque esté gordita o porque está más ¿sí?, ehhh sí, o sea, como que lo fui recuperando sola, entonces yo cada vez le decía a mi mamá ay cómpreme maquillaje entonces ah, cómpreme esto y me empecé a vestir bonito, a sentirme bonita ¿sí?, más que todo en la cara, porque ya mi cuerpo o sea no es que me encante, pero pues no me afecta tanto.
**A11**	For example, with all the problem I had last year, my safe space was volleyball, playing, you know what I mean? That's what gave me peace, it was the only thing that really helped me with my problems.	Por ejemplo, el año pasado sobre los problemas que tenía el año pasado, mi lugar seguro era voleibol, era jugar, ¿sí me hago entender?. Era lo que me daba paz, era lo único que me ha ayudaba con mis problemas.
**A14**	Like, rest, mental rest […] When I was at school, I barely had time for myself, to relax. However, this pandemic, even though the amount of rest time was exaggerated, also helped me a little to reflect on my behaviour, on who I was, and	Yo creo que el tema de, como el descanso, el descanso mental […] cuando estaba en el colegio yo casi no tenía tiempo para mí mismo, para relajarme. En cambio, esta pandemia, a pesar de que fue exagerada el tiempo

Quote ID	English Version (as published)	Original Spanish Version
	how I also wanted to give myself time for myself, which I didn't do before.	de descanso, me ayudó también un poco también a reflexionar sobre cómo era mi comportamiento, cómo era yo, cómo quiero también darme un tiempo para mí, que no me daba anteriormente.
**A22**	To be independent, not depend on anyone, be a source of pride for my parents. And in the future, to be able to be someone in life, you know? Like, for example, when I grow up, to be able to give to my parents and thank them and make them feel good, and for my part, to be on my own, to be able to do the things I like and all that, and to study what I like.	Ser independiente, no depender de nadie, ser un orgullo para mis papás. Y en el futuro, poder ser alguien en la vida, ¿sí?, o sea, por ejemplo, que yo en cuando crezca poder darles a mis papás y que como decirles gracias y hacerlos sentir bien y pues por mi parte que yo esté sola, que pueda hacer las cosas que me gusta y todo eso y estudiar lo que me gusta.
**A34**	And well, let's say I wouldn't if I could tell you whether I'm truly happy, because let' say sometimes I am, I won't lie, I am happy sometimes when we're joking around or laughing at some memes or silly things we see. But happy when I'm with someone or something, no, personally I wouldn't categorise myself as someone who's happy.	Y pues digamos que yo no sé, no sabría decirte que, si soy feliz, porque digamos que a veces sí, no te miento que soy feliz a veces cuando hacemos bromas o nos reímos de algunos chistes o memes que vemos. Pero feliz cuando, cuando estoy con alguna persona o algo, no, o personalmente no, para mí no me categorizaría como alguien feliz.
**A9**	And well, at first it was hard for me, because I was going through something difficult. I think at that time I reached a certain point of depression, because it really got to me, being locked up for so long, so much time, because those four walls, of course, drove me crazy and I was feeling bad every day… it even got to a point where for a week I didn't get out of bed, I was really not well, I hardly ate until… I said to myself, I can't let this defeat me because I have to, well, move up to tenth grade to be able to go on to eleventh.	Y pues al principio me dio duro, porque estaba pasando por algo difícil. Y creo que en ese tiempo llegué a tener cierto punto de depresión, porque me deprimió mucho, estar tanto, tanto tiempo encerrada porque cuatro paredes, claro, me enloquecí muchísimo y estaba mal yo todos los días… llegó incluso una semana en la cual no me paraba de la cama estaba mal, casi no comía hasta que… yo dije, no puedo dejar que eso me derrote

Quote ID	English Version (as published)	Original Spanish Version
		porque tengo que, pues pasar a décimo para pasar a 11.
**A42**	You see, I haven't really figured out what makes me happy. What I do know is that nothing truly makes me happy. Like, whatever I do, I don't feel satisfied with myself [...] I feel satisfied when I take a photo and it turns out nice and other like it on social media [...] but I've come to realise that' really bad, you know, doing things for other people instead of thinking about myself, so no, so far I haven't really figured out something that genuinely makes me happy.	Mira que no he sabido qué cosas me hacen feliz. Lo que sí sé es que nada me hace feliz. O sea, cualquier cosa que hago, no me siento satisfecha conmigo […] me siento satisfecha es tomarme una foto y que esa foto quede linda y que las demás personas le den me encanta en redes sociales […] pero pues eso he caído en cuenta que eso está muy mal, o sea hacer las cosas por las demás personas y no pensar en mí, entonces no, hasta el momento no es sabido hacer algo que me haga feliz a mí.
**A4**	I feel like everything is harder now [after the pandemic], uglier, like this is reality. Before, it was like "Oh, this is so nice," kind of like a little fairytale, so to speak. Now it's all just reality, like "Oh, life isn't like that," so why would I get my hopes up for something that's not going to happen? [...] the pandemic made us realise that very serious things were happening in the world.	Yo siento que ya, que ya todo es más duro, más feo, más, como que ya es la realidad. Antes era como Ay, no, esto es tan chévere, o sea, como un cuentico, por decirlo así, ahora como toda la realidad, como Ay, la vida no es así, entonces para que me voy a poner a ilusionarme con eso, si no me va a pasar […] la pandemia hizo como saber que en el mundo sí pasaban cosas muy fuertes.
**A42**	I hurt myself [...] I was in the bathroom having a shower and using those rough scrubbing pads, I'd grab my legs and scrub them really hard, and at that moment, I felt good. But now, thinking back, I know that wasn't okay at all. Because of that, I couldn't sleep, I just didn't sleep. I also started biting my lip, and I had this sore on my lip that bled, so I had to take antibiotics.	Me hice daño yo misma yo […] en el baño me estaba bañando y con esos estropajos duros que son, yo me cogía las piernas y me y me restregaba durísimo y me sentía en ese momento bien, que ahorita lo que pienso es que para para nada estaba bien y por lo que no podía dormir, no dormía. Eh, me empecé a morder el labio y yo tenía una pepa en el labio así y me sangraba y tuve que tomar antibiótico.
**A4**	There was a moment when I actually thought about ending my life because I	Yo hubo un momento donde sí pensé quitarme la vida por eso, porque yo dije,

Quote ID	English Version (as published)	Original Spanish Version
	said, I don't want to exist anymore, if I'm not good for my mum or my siblings, then I don't want to exist. But then she [my girlfriend] started telling me that I couldn't leave her alone and that it couldn't happen because it [being lesbian] was something that, eventually, would become normal for my mum and things like that… and I thought, well, I have to wait.	no quiero existir más porque, si no le hago bien a mi mamá, no le hago bien a mis hermanos, no quiero existir, pero pues ella [la novia] me empezó a decir que pues no la podía dejar sola y pues que eso no podía suceder porque era algo que en algún momento ya iba a ser normal para mi mamá y cosas así… y yo dije que pues toca esperar.
**A7**	Yes, that was also during the pandemic because, first, there was the pressure from school, they gave us too much homework [...] my parents always arguing, sometimes they fought really badly and said horrible things to each other [...] I did think about it once, at that time when everything was so awful, and I said to myself, I know how to do it, I'll grab a knife from the kitchen [...] I was actually planning it, thinking I could do this, until I realised, what am I doing? What am I thinking? Am I really thinking about dying? Once I did actually pick up a knife, and obviously I didn't go through with it, but I did end up leaving a mark...	Eso sí también fue en pandemia porque ya primero la presión del colegio, que dejaban demasiadas tareas [...] mis papás que siempre se peleaban, a veces se peleaban muy feo y se decían cosas horribles [...] yo sí llegué a pensarlo una vez, esa vez que estaba todo tan mal y yo dije yo sé cómo hacerlo y cojo un cuchillo de la cocina [...] yo lo estaba planeando, yo dije podría hacer esto hasta que me di cuenta que y dije ¿yo qué estoy haciendo?, ¿qué estoy pensando? o sea ¿me estoy pensando en morir? Una vez si alcancé a coger un cuchillo y pues obviamente no pude, pero sí me alcancé a dejar una marca...
**A17**	So, I started to think that I should value time better. Like, I've really begun to appreciate more the time I spend with someone I like, someone I get on well with. My perspective on life has really become clearer, it's about finding a purpose and at least fulfilling one dream.	Entonces yo me puse a pensar que debí valorar mejor el tiempo. Pues como como tal, comienza a valorar más el tiempo que paso por con alguien, que me agrada, alguien con quien me llevo bien. Mi perspectiva de la vida en realidad ya se esclareció, y es encontrar un objetivo, y cumplir al menos un sueño.
**A42**	I felt scared of not fitting in like everyone else seemed to. For example, with a group of friends, one would say something and the other would reply and they'd all laugh	Me sentía asustada de no encajar como encajaba todo el mundo. Por ejemplo, un grupo de amigos, uno decía tal cosa y el otro le respondía y se reían y todo,

Quote ID	English Version (as published)	Original Spanish Version
	and everything, or they'd just know what to say, like in a joke conversation, one would say something and the other would know exactly how to answer. My fear was not knowing how to reply, for example, saying something and no one laughing at it, or things like that, just not fitting in.	o sabían que decir, o sea en una charla de chiste, decía uno y el otro sabía que contestarle. Mi miedo era no saber contestar, por ejemplo, decir algo y que nadie se riera de eso chistoso que dije o, sí cosas así, no encajar.
**A42**	At that time, I loved it, and in the end, when the news started saying that we have to go back to school, well, I racked my brains and actually begged my mum to enrol me in a virtual school, like studying online, but not have to be around other people again. So, the lockdown wasn't so bad for me.	En ese momento me encantaba y a lo último cuando ya se empezó las noticias a decir ya tienen que volver a estudiar ya, o sea yo me maté mucho la cabeza y yo le rogué a mi mamá para que me metiera en un colegio virtual, una escuela virtual, o sea estudiar virtualmente, pero no volver a convivir con alguien. Entonces pues no fue tan malo para para mí la cuarentena.
**A37**	Sometimes I see other families that have an ugly stability, that is, dysfunctional families. But mine is not like that, mine is the complete opposite, you know? So sometimes when my mum sleeps in my bed, she tells me, "I love you, darling," and I just think, "My God, I really do feel good with what I have".	A veces yo veo familias, o sea que tienen una estabilidad fea, o sea, familias disfuncionales. En cambio, la mía no, la mía es todo lo contrario, ¿sí?, entonces mi mamá a veces duerme conmigo, me dice hija te amo y yo a veces digo, Dios mío, o sea sí me siento bien con lo que tengo.
**A11**	So when I called my dad [separated parents/divorced], I heard he was really unwell, I mean, really bad. So I told my mum, "Mum, my dad's sick and he's there all alone." And she said […] "You know what? We should bring him to our house." So my mum brought him to our place, she put him in my room, and since I had nothing to do all day, we weren't in school, just doing virtual classes, I was the one in charge of making his home remedies.	Entonces al llamar a mi papá lo escuché muy mal, o sea, mal, mal, mal. Entonces yo le dije: mami, mi papá que está enfermo y allá solo. Y me dijo, […] ¿sabe qué?, que se venga para acá para la casa, entonces mi mamá lo trajo para la casa de nosotros, lo puso en mi cuarto y le y yo como estaba todo el día, como no estudiábamos ni nada, sino virtual, yo era el encargado de hacerle la, los remedios.
**A9**	When I go out with them [my friends], believe me, I feel like I'm in a completely different environment than at home,	Porque yo salgo con ellos [mis amigos] y créeme que me siento en otro ambiente más diferente, que en mi

Quote ID	English Version (as published)	Original Spanish Version
	because they're just very different from the situation I'm living in at home. So, I feel good in that moment, and then when I go back home, it's like all that nice time I'd just spent is gone, because I return to my normal situation.	casa; porque ellos son más diferentes que en la situación que yo estoy viviendo en mi casa. Pero me sentía bien en ese momento y ya después llegaba como a la casa, entonces como que todo ese momento que había pasado bonito se iba, porque ya volvía a mi situación normal.
**A38**	There was a time when my mum lost her job [...] one time she couldn't pay a bill, and I said, well, it's okay, I'll sell my [video game] console, I wasn't really using it anyway. And I told her, like, it's fine, I'll sell it, and she was like, "No, no, how are we going to get out of this?" and so on… But I told her, it's alright, we'll sort it out later. So I sold it and paid the bill.	Es que un momento que a mi mamá perdió el trabajo [...] Y entonces, una vez que no podía este solventar un recibo y yo dije no, pues no, pues no importa, yo vendo la consola [de videojuegos], igual no la estaba usando y yo le dije pues bueno, yo la vendo y ella no, no, ¿qué vamos a salir de eso?, que no sé qué… Y yo le dije no pues no pasa nada. Después de ahí miramos, entonces la vendí, pagué el recibo.
**A8**	Once, for example, a friend told me he didn't have anything to eat for lunch, so I told my dad, and well, we weren't doing that well ourselves either, but my dad told me to invite him over to have lunch, at least so he has something warm and decent for that day. So he came, had lunch, thanked us; we also gave him, I think, 30,000 pesos [7 $ aprox.] so he could buy some more food, and we gave him some rice and beans too.	Una vez, por ejemplo, un amigo me dijo que no tenía que almorzar y yo le dije a mi papá y pues nosotros tampoco era que estuviéramos muy bien y mi papá me dijo que no, que lo invitara a almorzar, que por lo menos que por ese día tuviera algo seguro, calientico; y entonces el vino almorzó, nos dio las gracias; también les dimos, creo que 30000 pesos, para que pudiera comprar más comida y se llevó, creo que también un arroz y unos frijoles.
**A34**	Let's say that for a child, motivation doesn't really come from friends or girlfriends, it comes from the family. Maybe people should pay more attention to young people, because sometimes we feel really alone when we don't have family around, because that's the most important thing you should have throughout your life… family. And well, in my case, I don't have	Digamos que la motivación de niño no es tanto los amigos ni tampoco las novia, sino ya eso viene de familia. Tal vez que pusieran más atención a los jóvenes, porque a veces los jóvenes nos sentimos muy solos al no tener a un familiar, porque es lo principal que uno debe tener en todo en toda la vida… la familia. Y pues digamos que en mi caso

Quote ID	English Version (as published)	Original Spanish Version
	my family, they've never even asked about me. And that's what makes me feel sad sometimes, because I don't have a family. It's as if I were dead.	yo no tengo mi familia ya jamás se han preguntado por mí. Entonces eso es lo que a veces me pone triste porque yo no tengo familia, es como si estuviera muerto.
**A6**	Later on, you start to realise that sometimes you feel lonely, because even if you're with your family, it's not like you really share much with them. Well, back then [during the pandemic lockdown] I didn't really spend much time with my family, I was basically always on my own, just on my phone.	Pero luego uno se va dado cuenta que uno se siente sólo a veces porque pues, aunque uno esté con la familia, no es como que comparta demasiado, bueno en esos tiempos yo no compartía casi con mi familia, sino me la pasaba sólo técnicamente, en el teléfono.
**A2**	It was like I couldn't see them [friends], or things like that, so we started to stop talking, we kind of began to lose that friendship, you know? I mean, we were together all the time at school, but chatting wasn't the same, so we'd talk maybe once a month or so, and it just faded away…	Era como que no podía verlas, o esas cosas, entonces empezamos a dejar de hablar, empezamos como a perder esa amistad, ¿sí?, o sea estábamos todo el tiempo en el colegio, pero ya por chat no era igual, entonces hablábamos una vez al mes o así, y se fue perdiendo…
**A28**	One moment he [brother-in-law] is fine with you, and then he gets angry because you don't do him favours and things like that, even though he usually does nothing around the house, he just sleeps all day. He rarely goes out to work. And well, problems like that. Then, when he tried to cheat on my sister, he tried with my cousin, and I found out [...] I've tried to talk to my sister about it, but she's so in love with him that she doesn't believe me. Because of that, she stopped talking to me for about five months.	Con uno está bien en un momento, y después se pone bravo, porque uno no le hace favores y cosas así, aunque normalmente no hace nada en la casa, se la pasa es durmiendo [cuñado]. Rara vez sale a trabajar. Y pues problemas así y ya que como que intenta poner cacho mi hermana, le intentó poner cacho con mi prima y pues yo me di cuenta, [...] yo le he intentado cómo decir las cosas [a la hermana], pero como que está muy enamorada de él y no le cree a uno. Por base a eso me dejó de hablar como cinco meses, más o menos.
**A11**	And then more and more problems, and there's this thing when my mum gets angry, she says a word that destroys me, but she doesn't even realise it, she focuses	Y pues problema, más problema, más problema y que hay una cosa que mi mamá cuando está brava, me dice una palabra que me destruye, pero o sea

Quote ID	English Version (as published)	Original Spanish Version
	on saying that I'm useless, that I'm good for nothing. And that word, "useless," I feel is really destructive.	ella no se da cuenta, que es que ella se centra en que usted es inútil, usted no sirve para nada y esa palabra inútil, siento yo que es muy destructiva.
**A12**	Well, I hardly ever talked about that before because it was really hard to speak about. It turns out that when I was about, what, five or six years old, he was a very aggressive man, he used to hit my mum, he hit me too, he came home drunk, he [father] was incredibly unfaithful to my mum. My dad abandoned us, and the worst part was that he took everything, everything, leaving us sleeping on planks and cardboard.	Bueno, ese tema yo casi no lo hablaba anteriormente porque me daba muy duro hablar. Resulta que cuando tenía unos ¿qué?, cinco o seis años, él era un man altanero, le pegaba a mi mamá, me pegaba a mí, borracho llegaba, le era re infiel a mi mamá. Mi papá nos abandonó y lo más feo fue que él se llevó todo, todo, nos dejó durmiendo en tablas y en cartulinas, en cartulinas no, sino en cartón.
**A37**	I mean, it's complicated because if my mum doesn't pressure him, he doesn't give us anything, you know? And my mum doesn't really push him, in the sense of saying: "Hey, remember you have children, you've got two, I can't do everything on my own." Even though she doesn't earn much, she's the one who provides for the household	O sea, es complicado porque mi mamá si no lo presiona, no nos da, ¿sí? Entonces mi mamá no le hace la presión porque digamos es como "oye, recuerda que tienes hijos, tienes dos, yo no puedo sola todo", a pesar de que mi mamá no gana mucho ella sustenta la casa.
**A42**	She [my mum] argues with me a lot because she says I'm just like my dad and his family. […] Sometimes she says "You're rubbish, just like your dad. You… you hate me, you're going to leave me out on the street…" even though it's not true, I really do support her a lot when it comes to my dad.	Ella [mamá] pelea mucho conmigo porque dice que yo me parezco a mi papá y a la familia de él. [...] a veces me dice usted es una porquería, igual que su papá, usted, o sea, usted me odia, usted me va a dejar en la calle… usted, sabiendo que no, o sea yo, yo la respaldo mucho a ella con mi papá.
**A28**	We all started having clashes because we weren't used to seeing each other that much […] So, there were several arguments when we began spending more	Ya empezamos como a tener choques todos, porque no estábamos acostumbrados a vernos casi […] Entonces, hubo varias discusiones cuando empezamos a tener más

Quote ID	English Version (as published)	Original Spanish Version
	time together. And not being able to go out or do many things made it worse.	tiempo juntos. Y ya no poder salir, no poder hacer muchas cosas, por culpa de eso.
**Observation note (30.03.2023)**	Some teachers are talking about two girls who arranged to meet at the park to fight. Not only the two girls showed up, but also their friends. They started hitting each other; one girl left the other with a facial injury, and in response, she received such a strong blow that it fractured her arm. She is currently in the hospital.	Algunos profesores comentan que dos chicas se citaron en el parque para pelear. Al parque asistieron, no solo las dos chicas, sino las amigas de ambas. Comenzaron a darse golpes, una le dejó la cara herida a la otra, y como respuesta recibió un golpe tan fuerte que le fracturó un brazo y está en este momento en el hospital.
**Observation note (17.03.2023)**	Observation note (17.03.2023): A group of friends brought aguardiente (an alcoholic beverage) into the school. Some students ended up noticeably drunk, prompting the teachers to realise what was happening and call the parents to inform them. […] In a meeting between one of the students involved – who didn't appear to be particularly affected by alcohol – his parents, and the school coordinator, the father confronted the teenager, asking why he had to get himself into trouble if he knew that at home, whenever there's alcohol, they've already shared some with him. He added that at home, they've drunk together openly several times, so why would he go and create problems at school? He also said, "And why didn't you get drunk? Because at home we've taught you how to drink, haven't we?"	Un grupo de amigos entraron aguardiente (bebida alcohólica) al colegio, algunos adolescentes terminaron notablemente borrachos, por lo que los profesores se percataron de la situación y llamaron a los padres para informarles lo que había pasado [...] en una reunión entre uno de los estudiantes implicados, quien no se veía tan afectado por el consumo de alcohol, sus padres y el coordinador, el padre le reclama al adolescente diciéndole que, porque se tiene que meter en problemas, si en la casa él sabe que cuando hay trago ya le han compartido, que en la casa sin esconderse varias veces han tomado, entonces por qué tiene que buscarse problemas en el colegio. También le dice "y ¿usted por qué no se emborrachó?, ¡porque en la casa le hemos enseñado a tomar no cierto!"
**A42**	This past December I had a boyfriend, and I gave him everything, I told my family about him and everything, and then, on December 24th at nine at night, he asked for a break. He said I was insecure, that I didn't love him, and that I had made him	Ahorita en diciembre tuve un novio y a ese novio le entregue todo, o sea, le conté mi familia le y a lo último terminó el 24 de diciembre a las nueve de la noche pidiéndome tiempo. Y que yo era una insegura que si yo no me quería, no

Quote ID	English Version (as published)	Original Spanish Version
	feel really bad, and that was it, he let me go, I think that's why I fell into depression and got anxiety.	quería a él y que yo lo había hecho sentir muy mal y todo y me pidió tiempo y hasta ahí, o sea él me soltó y puso mi… Yo creo que por eso yo caí en depresión, me dio ansiedad.
**Observation note (31.10.2022)**	During the Halloween celebration, students from different grades organised an improvised party in one of the school's classrooms, where dancing became the central form of interaction. They wore revealing costumes, in line with current youth trends, and danced in pairs or trios using sensual body movements, typical of 'perreo' and other popular urban dacing styles and cultural trends. Physical closeness, bodily contact, and expressive movement were frequently observed among peers during interactions, particularly in shared activities and dance, reflecting patterns of social engagement.	Durante la celebración de Halloween, estudiantes de diferentes grados organizaron una fiesta improvisada en un salón del colegio, en la que el baile fue el eje central de la interacción. Llevaban disfraces reveladores, acordes con las tendencias juveniles actuales, y bailaban en parejas o tríos con movimientos corporales sensuales, típicos del perreo y otros estilos urbanos populares. La cercanía física, el contacto corporal y la expresividad del baile parecían representar una forma de exploración de la identidad, del deseo y del lugar que ocupan dentro del grupo de pares.
**C1**	So, another important thing is that here at school, the issue of child pornography became evident, for example, or boys who started watching platforms where they could sell sexual content [...] well, there were probably boys who thought: "Well, here I can make some money." However, there was also social pressure.	Entonces también algo importante es que acá en el Colegio, sí se evidenció el tema de la pornografía infantil, por ejemplo, o de chicos que empezaron a ver plataformas donde podían vender contenido sexual [...] pues habrá chicos que habrán dicho bueno acá puedo recoger algo de dinero, pero por otro lado también era como esa presión del contexto, digamos que social.
**C5**	Now, another thing that I think also increased during the pandemic was that we don't see each other, we don't have that contact, so we use the phone. And there's something that, I don't know if it's a coincidence, but right now it's completely out of control, the misuse of social media in terms of sexting, grooming, and all these situations where you see adolescents	Ahora bien, otra cosa que también creo yo que se incrementó en una pandemia fue como no tenemos, no nos vemos, no tenemos ese contacto, entonces usemos el celular. Y hay algo que, yo no sé si es casualidad, pero que está ahorita completamente disparado y ese mal manejo de las redes sociales en temas del sexting, del grooming, de

Quote ID	English Version (as published)	Original Spanish Version
	acting like it's completely normal without thinking about the risks involved.	todas estas situaciones que, pues uno ve que ahora ya los adolescentes practican como si fuera algo completamente normal sin pensar en los riesgos que hay.
**A10**	I became really obsessed, and that's why I couldn't manage it, because I obsessed over changing and wasn't satisfied with this reality. I kept asking, "Why do I exist here? Why can't I exist somewhere else?" But this year I found out that you can't do that, because you're putting pressure on your reality and your consciousness feels pressured and doesn't travel […] Why? For fun, because I want to experiment to see if it's real and with that you can mentally know many things.	Yo me obsesioné mucho, por eso yo no lo lograba, porque yo me obsesioné con cambiar y no me conformaba con esta realidad. Decía, ¿yo por qué existo aquí? ¿Por qué no puedo existir en otra?, pero me enteré en este año que eso no se podía hacer, porque estabas presionando a tu realidad y tú consciente se sentía presionado y no viajaba […]¿Por qué? Por gusto, porque quiero experimentar a ver si es real y con eso uno puede como mentalmente saber muchas cosas.
**A20**	I was just hangig out in my room, and he came in whileI was getting dressed and tried to force himself on me [...] my cousin was there, she was the one who came in and helped me [...] so my family took measures, like keeping a safe distance; well, social distancing and all that. And my mom said no, now she's more careful with me.	pues yo estaba normal en mi cuarto y él entró en un momento en el que yo me estaba vistiendo, e intentó por la fuerza [...] estaba mi prima, ella fue la que entró y como tal me ayudó [...] pues mi familia tomó las medidas de, pues la cuestión de que la sana distancia; pues sana distancia y todo eso. Y pues mi mamá dijo que no, mi mamá pues ahora, pues es como más cuidadosa conmigo.
**A8**	Sometimes there were people who asked for some groceries, they organised "solidarity teams" in the administration for those most in need in the housing complex. They registered apartments that said, "No, sorry, I need milk," "I need eggs," so sometimes donations were made; they took rice, beans, for those people.	Algunas veces sí había gente que pedía algunos víveres, hacían "donatones" en la administración para los más necesitados del conjunto, inscribían apartamentos que decían que no, que disculpen, que yo necesito leche, que yo necesito huevos, entonces algunas veces se iba a donar, se llevaban arroz, frijoles, para esas personas.

Quote ID	English Version (as published)	Original Spanish Version
**A30**	Next to my house they sell drugs, like marijuana and stuff. And, uh! Sometimes it really annoys me.	Al lado de mi casa, pues yo digo que son peor, porque eso no me gusta, por ejemplo, al lado de mi casa venden como que droga, sí marihuana y eso. Y ¡eh! a veces me da fastidio.
**A37**	Me without internet, I remember I didn't even have a phone either. My mum let me use hers, but since it was her work phone, she wouldn't really let me have it. My sister lent me a computer, but only for half a day because she needed it. So during the pandemic, I really fell behind, a lot, not just a little, I mean, my schoolwork was very poor.	Yo sin Internet, me acuerdo que tampoco tenía celular, mi mamá me dejaba el de ella, pero como era su me manera de trabajo no me lo dejaba yo… mi hermana me prestó un computador, pero me lo prestaba por día medio porque ella lo utilizaba. Entonces yo me descuidé un poco en pandemia, muchísimo, no, un poco no, muchísimo las tareas, o sea, la escolaridad muy baja.
**A37**	I'm really grateful to the school because during the pandemic, they gave vouchers to children from all schools. My brother got 50,000 [12 $ aprox.] and I got 50,000 [...] it would come in an email saying where to pick it up, like at the "name of the store," and 100,000 two years ago, that was a lot, I mean, it really was quite a bit [...] I'm very thankful because honestly, it helped us through a lot.	Al colegio le agradezco mucho porque en ese tiempo en pandemia dieron como unos bonos para para los niños de todos los colegios. Y a mi hermano le daban 50.000 y a mí me daban 50.000 [...] le llegaba en un correo y le decía que le tocaba en el "nombre del almacén" y con $100.000 hace dos años era harto, o sea, era bastante [...] le agradezco mucho porque de verdad no salvo de muchas cosas.
**A4**	I think I suffer from anxiety [...] my grandma told me to take this thing, it's like a pill, I don't know what it's called, but she gives it to me and it calms me down... Or she gives me very cold water with ice, really cold, and that helps too.	Yo digo que yo sufro de ansiedad [...] mi abuela me dijo que me tomara eso, es como una pasta, es que no sé cómo se llama, pero ella me la da y me calmó… O me da agua con hielo, pero muy fría, muy fría, también me ayuda.
**A8**	I used to spend a lot of time watching videos about self-love and how to improve yourself. Out of curiosity, I started watching to see what it was about. At that time, I wasn't feeling bad exactly, but I didn't feel great either. It was more like I	Me la pasada también viendo vídeos de amor propio, vídeos de cómo mejorarse uno mismo. Entonces, yo, por curiosidad, entre a ver qué era y entonces, pues en ese momento no era que me sentía mal, pero pues no me

Quote ID	English Version (as published)	Original Spanish Version
	was just existing […] Those videos taught me it’s okay to feel, it’s okay to have self-love, and it’s okay sometimes to feel low because that’s just part of who you are.	sentía tan bien. Era como pues existiendo ya […] entonces esos vídeos me enseñaron que está bien sentir, está bien tener amor propio, está bien algunas veces sentirse mal porque o sea, eso es parte de lo que es uno.
**A19**	Well, on Saturday… I mean, we go to church because on Saturdays they have activities for kids and at… at… 4:00 in the afternoon they have activities for young people.	Pues el sábado… o sea vamos a la iglesia porque el sábado hacen de niños y a las… a las… a las 4:00 de la tarde hacen de jóvenes.
